# GPR107 promotes autophagy secretion in psoriatic keratinocytes by inhibiting BECN1 K48-linked ubiquitination

**DOI:** 10.1038/s41419-026-09038-9

**Published:** 2026-06-26

**Authors:** Kainan Liao, Junyan Wang, Jinjin Chen, Dandan Zang, Tiantian Zhou, Nominzul Altankhuyag, Moru Puseletso, Jing Wang, Chunlin Cai, Fusheng Zhou, Deping Xu, Haisheng Zhou

**Affiliations:** 1 Department of Biochemistry and Molecular Biology, Anhui Medical University (AHMU), Hefei, China; 2 Department of Gastroenterology, The First Affiliated Hospital of AHMU, Hefei, China; 3 Clinical Laboratory, The Second Affiliated Hospital of AHMU, Hefei, China; 4 Center for Scientific Research, AHMU, Hefei, China; 5 Undergraduate Student of The 2023 Cohort at The First Clinical Medical College, AHMU, Hefei, China; 6 Department of Pathophysiology, AHMU, Hefei, China; 7 Key Laboratory of Dermatology, AHMU & Ministry of Education, Hefei, China; 8 Clinical Laboratory, The Second People’s Hospital of Hefei & The Affiliated Hefei Hospital of AHMU, Hefei, China

**Keywords:** Psoriasis, Experimental models of disease

## Abstract

Psoriasis is a chronic inflammatory skin disease driven by excessive proliferation and aberrant differentiation of keratinocytes. Autophagy-based unconventional secretory pathway (secretory autophagy) plays key roles in regulating cell proliferation and autosecretion in psoriatic keratinocytes; however, their upstream regulators remain poorly defined. The orphan G protein-coupled receptor 107 (GPR107) mediates signal transduction via clathrin-dependent endocytosis. Here, we report that upregulation of GPR107 in psoriatic keratinocytes promotes both cell proliferation and the secretion of chemokines and antimicrobial peptides through BECN1-dependent autophagy. Mechanistically, internalized GPR107 activates the β-arrestin/ERK/NF-κB pathway. This activation not only drives the direct transcription of inflammatory factors but also negatively regulates the expression of E3-ubiquitin ligase CUL3. The reduction of CUL3 decreases K48-linked ubiquitination at K206 of BECN1, preventing its proteasomal degradation. Stabilization of BECN1 facilitates secretory autophagy in psoriatic keratinocytes, further enhancing their proliferation and inflammatory responses. These findings highlight a novel function of GPR107 in psoriasis, and suggest that the integrated β-arrestin/ERK/NF-κB/CUL3/BECN1 axis may serve as a potential therapeutic target for this disease.

## Introduction

Psoriasis is a chronic immune-mediated skin disease affecting 2–3% of the global population [1, 2] characterized by erythema, epidermal thickening, hyperkeratosis, and immune cell infiltration [3–5]. Keratinocytes play a central pathogenic role, driving epidermal hyperplasia, aberrant keratinization, and pro-inflammatory signaling [6–8]. In psoriatic lesions, pro-inflammatory cytokines stimulate keratinocytes to produce chemokines (e.g., CCL20, CXCLs) and antimicrobial peptides (e.g., S100A), which in turn recruit IL-17A-producing Th17 cells and neutrophils, reinforcing the IL-23/IL-17A axis and sustaining chronic inflammation [9–13]. Consequently, autosecretion in psoriatic keratinocytes contributes to inflammatory responses and promotes the development and persistence of psoriatic lesions.

Macroautophagy (autophagy) is a crucial intracellular catabolic process that degrades cellular constituents to maintain homeostasis [14, 15]. It plays a central role in cellular metabolism, innate and adaptive immunity, and antigen presentation [16, 17], making it a potential therapeutic target for several autoimmune diseases [18]. BECN1 (BECLIN1) serves as a molecular platform which interacts with a multitude of proteins-including PI3K, BCL2, ATG14, UVRAG, and RUBCN-to regulate autophagic flux [19]. The BECN1 complex is therefore widely recognized as a key regulator of autophagy-commonly referred to as BECN1-dependent autophagy-orchestrating both stimulatory and inhibitory protein partners within its interactome. Emerging evidence suggests that the function of BECN1-dependent autophagy extends beyond canonical autophagy and can influence inflammatory processes [19, 20]. In particular, autophagy-based unconventional secretion, also known as secretory autophagy, represents a newly characterized function of autophagy that contributes to tissue development and remodeling, tumorigenesis, aging, and notably, inflammation [21–23]. This discovery broadens the functional scope of autophagy from maintaining intracellular homeostasis to regulating extracellular signaling. In psoriasis, autophagy-related markers are upregulated and functionally active in both patient-derived keratinocytes and murine models [24, 25]. Interestingly, recent research by Zhen Wang et al. demonstrated that an autophagy-based unconventional secretory pathway (secretory autophagy), dependent on autophagy-related 5 (ATG5) and Golgi reassembly stacking protein 2, promotes psoriasiform inflammation in keratinocytes [26]. However, the mechanisms regulating secretory autophagy in psoriatic keratinocytes remain unclear.

G protein-coupled receptor 107 (GPR107), an orphan receptor belonging to the GPCR superfamily, was initially identified from a human lung cDNA library [27]. It is expressed in multiple tissues, including the brain, heart, liver, and kidneys [27, 28]. Genetic ablation of *Gpr107* results in embryonic lethality between 11.5 and 12.5 days post coitus, indicating that GPR107 serves as a key regulator of embryonic development [29]. Functionally, GPR107 promotes clathrin-dependent transferrin endocytosis and recycling in mouse embryonic fibroblasts [29]. Previous studies have demonstrated that internalized GPR107, acting through clathrin-mediated endocytosis (CME) and mediating ERK/NF-κB signaling, plays a critical role in regulating collagen IV internalization in the glomerular filtration membrane associated with diabetic nephropathy, as well as in breast cancer, where it promotes cell migration and invasion [30, 31]. Notably, both endocytosis and autophagy are membrane-vesicle-based cellular pathways, and growing evidence indicates that the function of BECN1 complex is not restricted to autophagy but it can be involved in phagocytosis and endocytosis [19, 20, 32]. Therefore, we hypothesized that GPR107 may mediate signaling to regulate BECN1-dependent autophagy secretion in psoriatic keratinocytes and be involved in the pathogenesis of psoriasis.

In this study, we found that upregulation of GPR107 promotes the proliferation and autosecretion of psoriatic keratinocytes through BECN1-dependent autophagy. Mechanistically, GPR107 activates the β-arrestin/ERK/NF-κB signaling pathway to suppress the expression of the E3 ligase CUL3, thereby decreasing K48-linked ubiquitination of BECN1 and reducing its degradation. These findings provide new insights into the molecular mechanisms of psoriasis and suggest potential therapeutic targets for its treatment.

## Materials and methods

### Patients and skin specimens

All participants in this study, including 5 patients with psoriasis and 5 healthy donors, were recruited from the Department of Dermatology, First Affiliated Hospital of AHMU. Skin biopsies (6 mm) were obtained from lesional sites of psoriasis patients, while biopsies from non-inflamed skin of healthy donors served as controls.

### Mice, imiquimod (IMQ)-induced psoriasis model, and treatment

C57BL/6 J mice were obtained from Nanjing Gem Pharma Co. (Nanjing, China). *Gpr107-floxed* mice (*Gpr107*^*f/f*^*)* and *Krt14cre* mice, both on a C57BL/6 background, were purchased from Cyagen Biosciences (Wuhan, China). The generation and identification of keratinocyte-specific *Gpr107* knockout mice are described in Fig. S1. The establishment of IMQ-induced psoriasis models and the knockdown of *Gpr107* expression in IMQ-induced psoriasiform lesions were performed as previously reported [33, 34] and are detailed in the *Supplementary Information*.

### Other materials and methods

A detailed description of all other experimental procedures, including cell culture and treatments, histological analysis, protein expression detection (Western blotting, immunohistochemistry, immunofluorescence, and co-immunoprecipitation), mRNA detection and analysis, as well as statistical analysis, is provided in the *Supplementary Information*.

### Ethics approval and consent to participate

Human biopsy collection was approved by the Biomedical Ethics Committee of AHMU (approval no. 20200290) and conformed to the ethical standards for medical research involving human subjects, as laid out in the 1964 Declaration of Helsinki and its later amendments. All animal experiments followed the WMA Statement on animal use in biomedical research and approved by the Experimental Animal Ethics Committee of AHMU (approval no. LLSC20200007). Written informed consent was obtained from all participants.

## Results

### Upregulation of GPR107 contributes to the proliferation and autocrine secretion of psoriatic keratinocytes

**Fig. 1:**
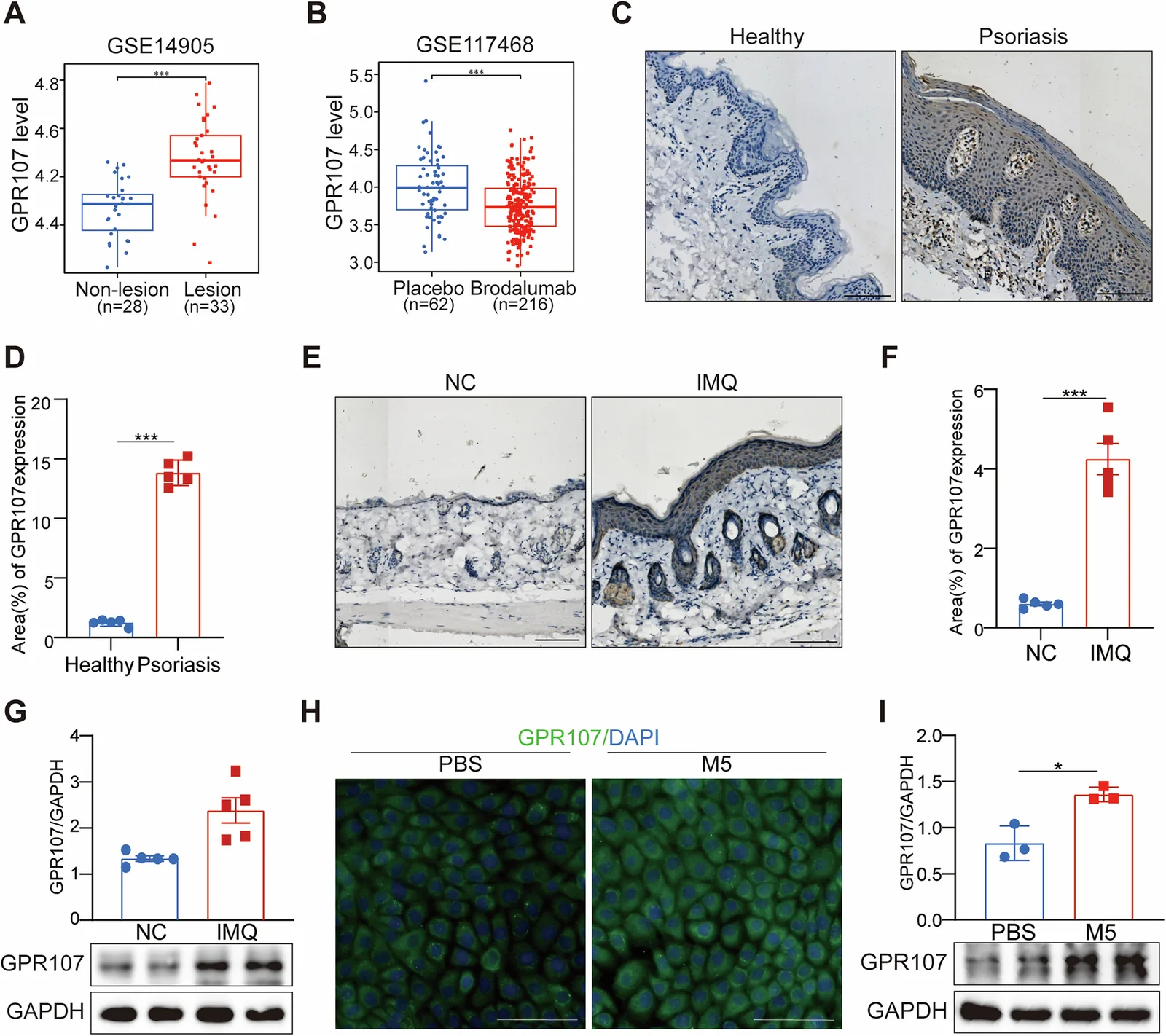
GPR107 upregulation in human psoriatic epidermis, IMQ-induced psoriasiform skin, and M5-induced cellular models. **A** *GPR107* mRNA levels in lesional versus non-lesional psoriatic skin. Data were obtained from the Gene Expression Omnibus (GEO) database (GSE14905). **B** *GPR107* mRNA levels in psoriatic patients treated with Placebo or Brodalumab (GEO database GSE117468). **C**, **D** Immunohistochemical analysis of GPR107 in healthy control and psoriatic skin. Scale bars = 100 μm. Data are presented as mean ± SD (*n* = 5), ***p < 0.001. **E**, **F** Immunohistochemistry analysis of GPR107 expression in mouse control skin (vaseline-treated, NC) and IMQ-induced psoriasiform lesions (IMQ). Scale bars = 100 μm. Data are presented as mean ± SD (*n* = 5), ***p < 0.001. **G** Western blot analysis of GPR107 protein expression in vaseline-treated mouse skin (NC) and IMQ-induced psoriasiform lesions (IMQ). Data are presented as mean ± SD (*n* = 5), *p < 0.05. **H** Immunofluorescence detection of GPR107 in HaCaT cells treated with PBS or M5 for 24 h. Scale bars = 20 μm. **I** Western blot analysis of GPR107 protein expression in NHEK cells treated with PBS or M5 for 24 h. Data are presented as mean ± SD (*n* = 3), *p < 0.05.

To investigate the association between psoriasis and GPR107, we first analyzed the Gene Expression Omnibus dataset GSE14905 and GSE117468. Our analysis revealed that GPR107 mRNA levels were significantly elevated in psoriatic lesions compared to non-lesional skin (Fig. 1A), and were markedly reduced after Brodalumab treatment (Fig. 1B). We next examined GPR107 expression in human skin biopsies, observing a marked upregulation in psoriatic lesions via immunohistochemical staining (Fig. 1C, D and S1A). Successful modeling of IMQ-induced psoriasis was characterized by the downregulation of loricrin in IHC analysis (Fig. S1B) and the upregulation of *Il-17*, *S100a7*, and β4-defensin in RT-qPCR (Fig. S1C). Consistently, immunohistochemical staining in an IMQ-induced mouse model of psoriasis showed increased epidermal expression of GPR107 (Fig. 1E, F and S1D). Western blot analysis further confirmed significant upregulation of GPR107 in IMQ-induced psoriatic mice compared with controls (Fig. 1G). To evaluate GPR107 expression in an in vitro cellular model of psoriasis, we stimulated HaCaT cells with the M5 cytokine mixture (comprising IL-17A, IL-22, IL-1α, Oncostatin M, and TNF-α, each at a final concentration of 10 ng/mL) to induce psoriasis-like inflammation. Immunofluorescence analysis confirmed a pronounced increase in GPR107 expression in M5-treated HaCaT cells (Fig. 1H). Similarly, elevated GPR107 expression was validated in primary normal human epidermal keratinocytes following M5 treatment (Fig. 1I).

To investigate the roles of GPR107 in psoriasis, keratinocyte-specific *Gpr107* knockout mice were generated by crossing *Gpr107*^*f/f*^ mice with *Krt14-Cre* transgenic mice. The resulting mice, bearing selective deletion of *Gpr107* in keratinocytes (*Krt14*^*Cre/+*^*-Gpr107*^*f/f*^), were compared with their littermate controls (*Krt14*^*+/+*^*-Gpr107*^*f/f*^ *mice*) to evaluate the impact of GPR107 deficiency on psoriasis pathogenesis (Fig. S2A). Keratinocyte-specific deletion of *Gpr107* was confirmed by PCR analysis of genomic DNA isolated from mouse tails (Fig. S2B–D), and the loss of protein expression was further validated (Fig. S2E).

**Fig. 2:**
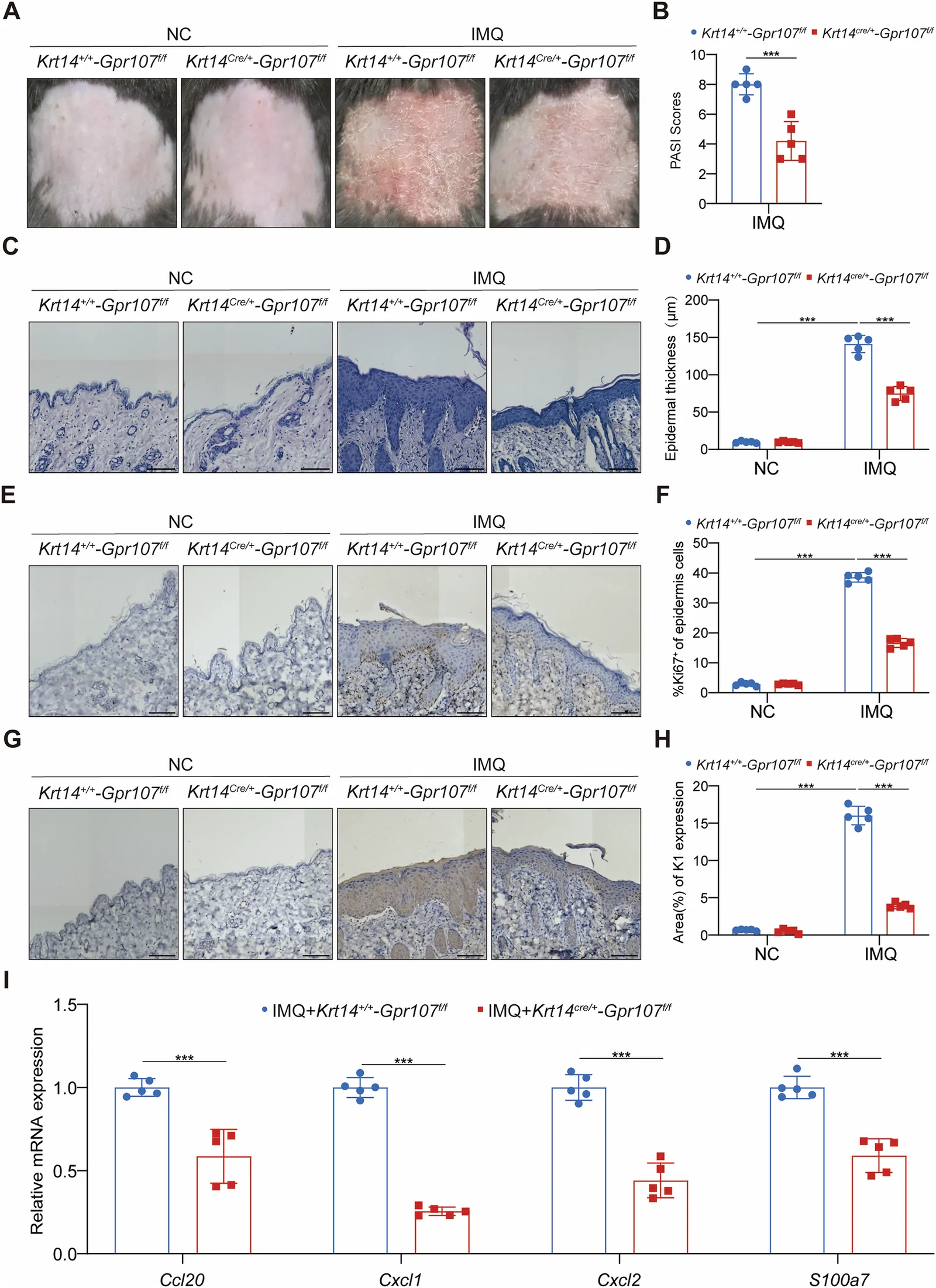
Depletion of GPR107 in keratinocytes ameliorates IMQ-induced psoriasiform phenotype. **A** Representative images of dorsal skin from mice treated with vaseline (NC) and mice treated with IMQ for 6 days. **B** Analysis of PASI scores in 6-day IMQ-induced psoriasiform lesions. Data are presented as mean ± SD (*n* = 5), ***p < 0.001. **C** Representative H&E-stained histological sections of dorsal skin from *Krt14*^*+/+*^*-Gpr107*^*f/f*^ and *Krt14*^*Cre/+*^*-Gpr107*^*f/f*^ mice treated with vaseline (NC) or IMQ for 6 days. Scale bars = 100 μm. **D** Quantitative analysis of epidermal thickness. Data are presented as mean ± SD (*n* = 5). ***p < 0.001. **E**, **F** Immunohistochemistry analysis of Ki-67-positive cells in the skin of *Krt14*^*+/+*^*-Gpr107*^*f/f*^ and *Krt14*^*Cre/+*^*-Gpr107*^*f/f*^ mice treated with vaseline (NC) or IMQ for 6 days. Scale bars = 100 μm. Data are presented as mean ± SD (*n* = 5). ***p < 0.001. **G**, **H** Immunohistochemistry analysis of keratin 1 (K1) expression in dorsal skin of *Krt14*^*+/+*^*-Gpr107*^*f/f*^ and *Krt14*^*Cre/+*^*-Gpr107*^*f/f*^ mice treated with vaseline (NC) or IMQ for 6 days. Scale bars = 100 μm. Data are presented as mean ± SD (*n* = 5). ***p < 0.001. **I** qRT-PCR analysis of *Ccl20*, *Cxcl1*, *Cxcl2*, and *S100a7* mRNA levels in the skin of *Krt14*^*+/+*^*-Gpr107*^*f/f*^ and *Krt14*^*Cre/+*^*-Gpr107*^*f/f*^ mice treated with IMQ for 6 days. Data are presented as mean ± SD (*n* = 5). *** *p* < 0.001.

Although global deletion of *Gpr107* results in an embryonic lethal phenotype [29], no obvious changes in epidermal morphology or homeostasis were observed in the keratinocyte-specific *Gpr107* knockout mice. Upon induction of a psoriasis-like inflammation model by topical application of IMQ on the dorsal skin of mice, psoriasiform lesions were observed in both *Krt14*^*Cre/+*^*-Gpr107*^*f/f*^ mice and *Krt14*^*+/+*^*-Gpr107*^*f/f*^ mice. Notably, *Krt14*^*+/+*^*-Gpr107*^*f/f*^ mice exhibited more severe cutaneous injury than *Krt14*^*Cre/+*^*-Gpr107*^*f/f*^ mice (Fig. 2A). The Psoriasis Area and Severity Index (PASI) score was significantly lower in *Krt14*^*Cre/+*^*-Gpr107*^*f/f*^ mice compared with *Krt14*^*+/+*^*-Gpr107*^*f/f*^ mice (Fig. 2B). Consistently, H&E staining revealed that epidermal thickness in the dorsal skin of *Krt14*^*+/+*^*-Gpr107*^*f/f*^ mice was greater than that in *Krt14*^*Cre/+*^*-Gpr107*^*f/f*^ mice (Fig. 2C, D). Immunohistochemical analysis showed a significant reduction in the number of Ki-67-positive keratinocytes in IMQ-treated *Krt14*^*Cre/+*^*-Gpr107*^*f/f*^ mice compared with *Krt14*^*+/+*^*-Gpr107*^*f/f*^ mice (Fig. 2E, F and S3A). Similarly, decreased K1 expression was observed in the *Krt14*^*Cre/+*^*-Gpr107*^*f/f*^ mice following IMQ treatment (Fig. 2G, H and S3B). Importantly, the expression of neutrophil chemokines (*Ccl20, Cxcl1*, and *Cxcl2*) and the antimicrobial peptide *S100a7* in skin lesions was lower in *Krt14*^*Cre/+*^*-Gpr107*^*f/f*^ mice than in *Krt14*^*+/+*^*-Gpr107*^*f/f*^ mice (Fig. 2I). Together, these findings provide important insights into the potential role of GPR107 in modulating not only keratinocyte proliferation but also inflammation in response to IMQ treatment.

### GPR107 drives secretory autophagy in psoriatic keratinocytes through BECN1 upregulation

**Fig. 3:**
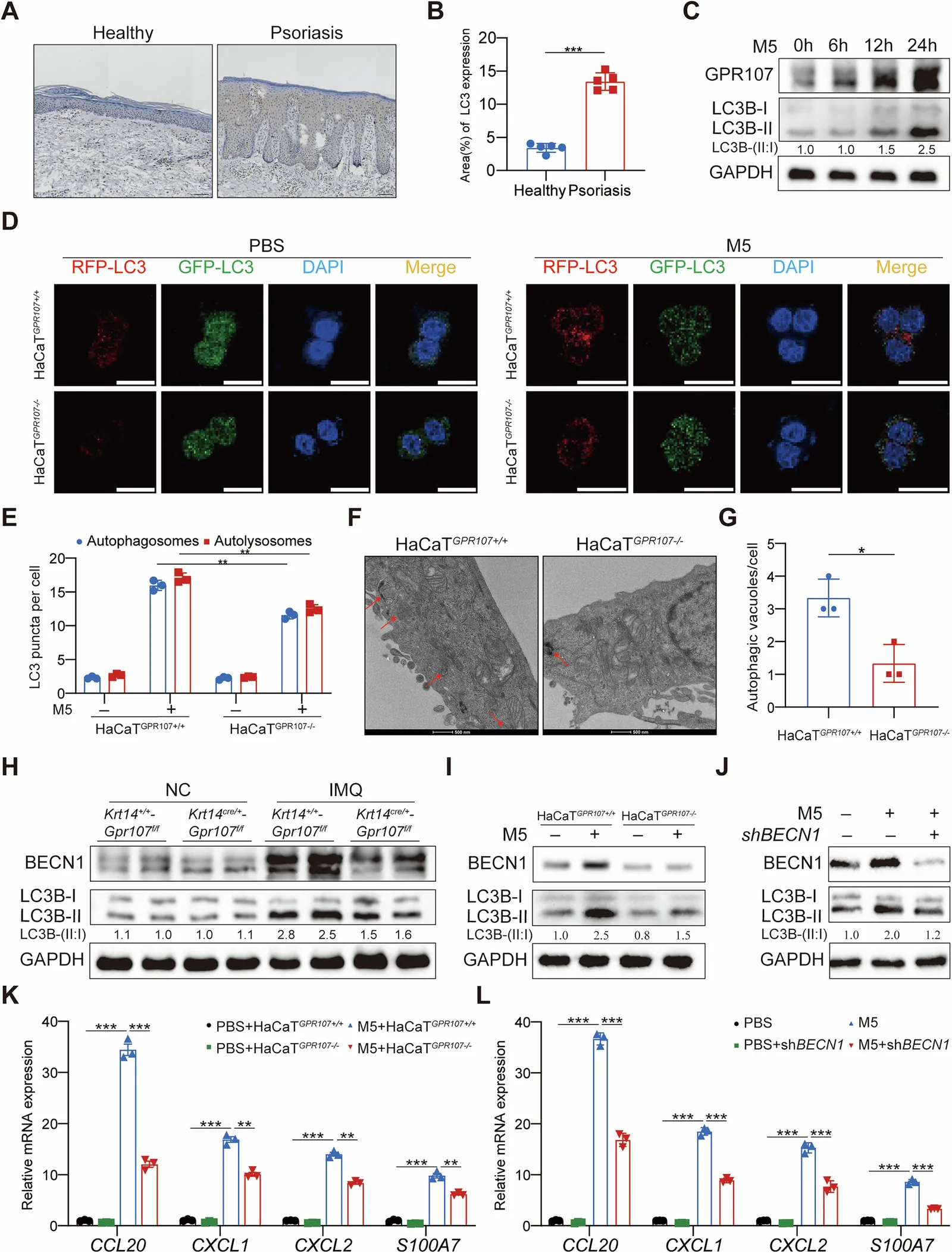
GPR107 regulates autophagy-based secretion in a BECN1-dependent manner. **A**, **B** Immunohistochemistry analysis of LC3B expression in skin of healthy controls and psoriasis patients. Scale bar = 100 μm. Data are presented as mean ± SD (*n* = 5), ***p < 0.001. **C** Western blot analysis of GPR107, LC3B-II, and LC3B-I protein levels in HaCaT cells treated with M5 for 0, 6, 12, and 24 h. The LC3B-II/LC3B-I ratio represents the LC3B-II to LC3B-I ratio. **D** Representative fluorescent images of LC3B puncta were captured. Cells were transfected with the mRFP-GFP-LC3B vector. After 24 h, cells were treated with PBS or M5 for another 24 h. Yellow puncta indicates autophagosomes, and red puncta indicates autolysosomes. Scale bars = 20 μm. **E** Fluorescent punctas quantification analysis. The mean number of autolysosomes and autolysosomes per cell is plotted (*n* = 3). **p < 0.01. **F** Representative electron micrographs of HaCaT^G*PR107*+/+^ and HaCaT^*GPR107*-/-^ cells treated with M5 for 24 h. Scale bars = 500 nm. **G** Autophagosome quantification in HaCaT^G*PR107*+/+^ and HaCaT^*GPR107*-/-^ cells treated with M5 for 24 h. Data are presented as mean ± SD (*n* = 3). *p < 0.05. **H** Western blot analysis of BECN1 and LC3II/I protein levels in the skin of *Krt14*^*+/+*^*-Gpr107* ^*f/f*^ and *Krt14* ^*Cre/+*^*-Gpr107* ^*f/f*^ mice treated with vaseline (NC) or IMQ for 6 days. The LC3II/I ratio (LC3II to LC3I) was determined. **I** Western blot analysis of BECN1 and LC3II/I protein levels in HaCaT^G*PR107*+/+^ and HaCaT^*GPR107*-/-^ cells treated with or without M5 for 24 h. **J** Western blot analysis of M5-induced LC3II/I ratio alterations in HaCaT cells with *BECN1* knockdown. **K** qRT-PCR analysis of *CCL20*, *CXCL1*, *CXCL2*, and *S100A7* mRNA levels in HaCaT^G*PR107*+/+^ and HaCaT^*GPR107*-/-^ cells treated with PBS or M5 treatment for 24 h. Data are presented as mean ± SD (*n* = 3). **p < 0.01, ***p < 0.001. **L** qRT-PCR analysis of *CCL20*, *CXCL1*, *CXCL2*, and *S100A7* mRNA levels in HaCaT cells following M5 stimulation and/or *BECN1* knockdown (sh*BECN1*). Data are presented as mean ± SD (*n* = 3). ***p < 0.001.

Since autophagy modulates the secretion of inflammatory factors, chemokines, and antimicrobial peptides through its secretory autophagy [26], it remains to be determined whether GPR107-modulated inflammation is associated with this mechanism in psoriasis. In fact, LC3B was notably elevated in psoriatic lesions compared to normal epidermis (Figs. 3A, B and S4A). To further investigate the effect of GPR107 on autophagy, we treated keratinocytes with M5, and observed that as GPR107 expression increased with prolonged M5 treatment, the LC3B-II/LC3B-I ratio also increased (Fig. 3C). Subsequently, *GPR107*-knockout HaCaT cells were generated for autophagic flux analysis (Fig. S2F). This analysis revealed that, in M5-stimulated keratinocytes, the formation of both autophagosomes (yellow puncta, RFP^+^GFP^+^) and autolysosomes (red puncta, RFP^+^GFP^-^) was significantly reduced in the absence of GPR107 (Fig. 3D, E). Electron microscopy ultrastructural analysis also showed that the number of autophagic vacuoles per cell significantly decreased in M5-treated *GPR107*-knockout keratinocytes compared with M5-treated normal keratinocytes (Fig. 3F, G).

Importantly, analysis of LC3B expression in the IMQ-induced psoriasiform lesions showed that the LC3B-II/LC3B-I ratio was markedly decreased in the skin tissues of *Krt14*^*Cre/+*^*-Gpr107*^*f/f*^ mice compared with *Krt14*^*+/+*^*-Gpr107*^*f/f*^ mice (Fig. 3H). These data indicated that GPR107 deficiency in the IMQ-induced psoriasiform lesions suppressed autophagy. Consistently, BECN1 expression, which was induced by IMQ in psoriasiform lesions, was downregulated in *Krt14*^*Cre/+*^*-Gpr107*^*f/f*^ mice. Knockout of *GPR107* in M5-treated keratinocytes also led to a decrease in the LC3B-II/LC3B-I ratio and in BECN1 protein levels (Fig. 3I). Furthermore, knockdown of *BECN1* in keratinocytes under M5 stimulation resulted in a reduced LC3B-II/LC3B-I ratio (Fig. 3J). Collectively, these findings suggest that GPR107 is associated with the regulation of autophagy in psoriatic keratinocytes *via* BECN1 expression.

To assess the function of GPR107 in secretory autophagy, the levels of chemokines (*CCL20*, *CXCL1*, and *CXCL2*) and the antimicrobial peptide *S100A7* were determined in M5-stimulated keratinocytes. M5 stimulation robustly induced the expression of these chmokines and *S100A7* in HaCaT cells. Conversely, pharmacological blockades of autophagy using bafilomycin A1 (BafA1) significantly downregulated the mRNA levels of these chemokines and *S100a7* (Fig. S4B). Interestingly, upon M5 stimulation, the expression of these chemokines and the antimicrobial peptide was significantly decreased in HaCat^*GPR107*-/-^ cells compared with HaCat^*GPR107*+/+^ cells (Fig. 3K and S5A). Furthermore, BECN1 knockdown significantly attenuated M5-induced expression in these cells (Fig. 3L). Therefore, these findings indicate that GPR107 may contribute to psoriasis pathogenesis, potentially through modulation of BECN1-mediated secretory autophagy.

### GPR107-induced BECN1 upregulation depends on CUL3-mediated ubiquitination

**Fig. 4:**
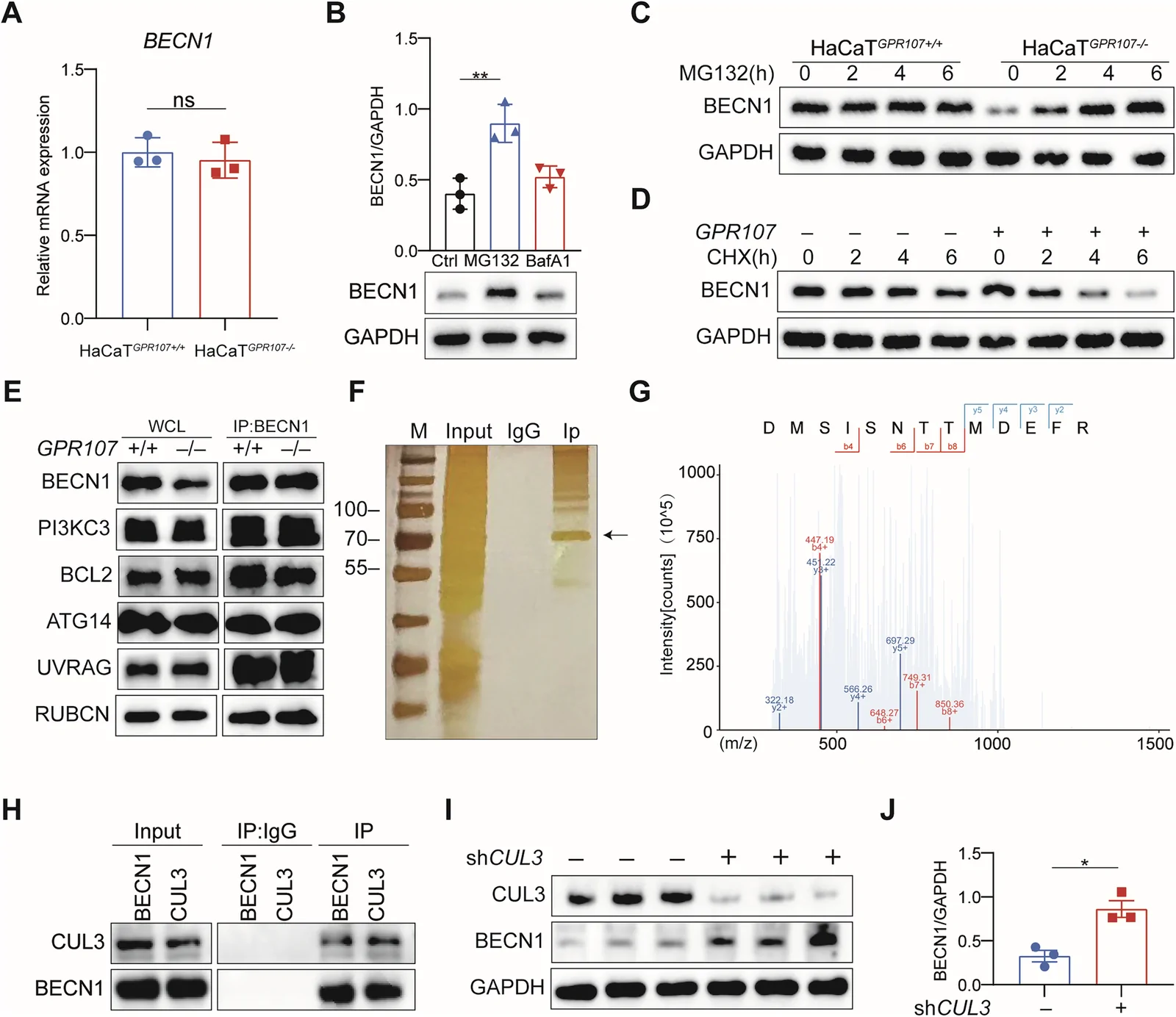
GPR107 facilitates BECN1 degradation *via* CUL3-dependent proteasomal pathway. **A** qRT-PCR analysis of *BECN1* mRNA levels in HaCaT^G*PR107*+/+^ and HaCaT^*GPR107*-/-^ cells. Data are presented as mean ± SD (*n* = 3). “ns” indicates no significant difference. **B** Western blot analysis of BECN1 protein levels in HaCaT cells treated with MG132 (10 µM) or BafA1 (100 nM) for 24 h. Data are presented as mean ± SD (*n* = 3). **p < 0.01. **C** Western blot analysis of BECN1 protein levels in HaCaT^G*PR107*+/+^ and HaCaT^*GPR107*-/-^ cells treated with MG132 (10 µM) for 0, 2, 4, and 6 h. **D** Western blot analysis was performed to examine the levels of BECN1 protein in HaCaT cells and Flag*-GPR107*-transfected HaCaT cells treated with CHX (100 μg/ml) for 0, 2, 4, and 6 h. **E** Co-immunoprecipitation (Co-IP) and Western blot analysis of BECN1 protein and its complexes in HaCaT^G*PR107*+/+^ and HaCaT^*GPR107*-/-^ cells. PIK3C3, class III mammalian phosphatidylinositol-3 kinase; BCL2, B-cell lymphoma 2; UVRAG, ultraviolet irradiation resistance-associated gene; RUBCN, RUN domain and cysteine-rich containing, becn1-interacting protein (Rubicon). **F** Proteins co-immunoprecipitated with BECN1 from HaCaT cells treated with MG132 (10 µM) for 6 h were visualized by silver staining. The arrow indicates a potential target protein selected for subsequent MS identification. **G** MS analysis identified CUL3 peptide fragments from the potential target protein co-immunoprecipitated with BECN1. **H** Co-IP and Western blot analysis of BECN1 and CUL3 in HaCaT cells treated with MG132 (10 µM) for 6 h. Rabbit IgG was used as a negative control. **I** Western blot analysis of BECN1 expression in HaCaT cells with *CUL3* knockdown. **J** Western blot analysis of BECN1 expression in HaCaT cells with *CUL3* knockdown. Data are presented as mean ± SD (*n* = 3), *p < 0.05.

To determine whether GPR107-induced upregulation of BECN1 occurs at the mRNA or protein level, we first measured *BECN1* mRNA levels and observed no significant difference between normal keratinocytes and *GPR107-*knockout keratinocytes (Fig. 4A). Thus, GPR107-mediated upregulation of BECN1 likely involves post-translational mechanisms affecting protein degradation. To further examine BECN1 degradation, we utilized specific inhibitors targeting major protein degradation routes in mammalian cells, namely the ubiquitin-proteasome system (UPS) and the autophagy-lysosomal pathway. As shown in Fig. 4B, the proteasome inhibitor MG132 elevated BECN1 protein levels in keratinocytes, whereas the lysosomal inhibitor Bafilomycin A1 (BafA1) had no significant effect. Importantly, in *GPR107*-knockout keratinocytes, MG132 treatment resulted in a dose-dependent increase in BECN1 protein levels (Fig. 4C). In addition, overexpression of GPR107 reduced the half-life of BECN1 protein, as shown by Cycloheximide (CHX) chase assays (Fig. 4D). As a whole, these findings indicate that GPR107 appears to modulate the stability of BECN1 *via* the UPS pathway.

Considering the regulation of the BECN1 complex-which initiates autophagosome formation via PIK3C3/Vps34, ATG14, and UVRAG, and is negatively regulated by RUBCN and BCL2, we aimed to determine whether GPR107, in addition to promoting BECN1 degradation, also modulates the assembly and activity of this complex. GPR107 deficiency did not affect the association between BECN1 and its binding partners (PIK3C3/Vps34, ATG14, UVRAG, BCL2, or RUBCN) (Fig. 4E), suggesting that GPR107 inhibits autophagic flux specifically by mediating BECN1 degradation, rather than by disrupting BECN1 complex assembly. We next performed immunoprecipitation assays targeting BECN1 to explore the specific mediators involved in GPR107-induced BECN1 degradation. Mass spectrometry (MS) analysis of the BECN1 immunoprecipitates identified several interacting proteins (Fig. 4F). Cullin (CUL3), a scaffold component of the E3 ubiquitin ligase, was selected for further characterization (Fig. 4G, H). In particular, increased BECN1 protein levels were observed in CUL3-knockdown keratinocytes (Fig. 4I, J), suggesting that CUL3 appears to regulate BECN1 degradation by modulating its ubiquitination.

### BECN1 undergoes K48-ubiquitination mediated by CUL3

**Fig. 5:**
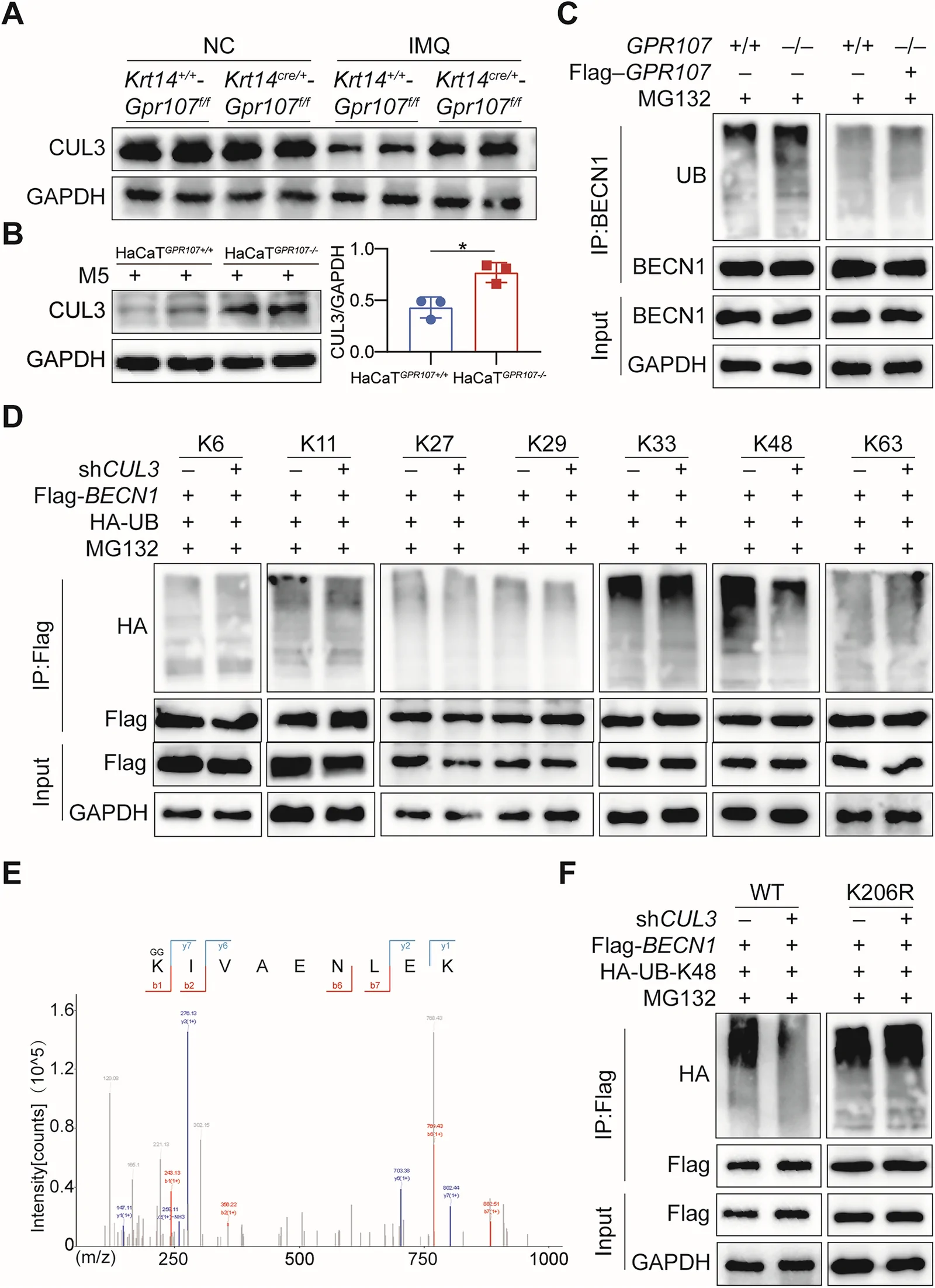
Increased CUL3 promotes K48-linked ubiquitination of BECN1 in GPR107-deficient psoriatic keratinocytes. **A** Western blot analysis of CUL3 expression in the skin of *Krt14*^*+/+*^*-Gpr107*^*f/f*^ and *Krt14* ^*Cre/+*^*-Gpr107*^*f/f*^ mice treated with vaseline (NC) or IMQ for 6 days. **B** Western blot analysis of CUL3 expression in HaCaT^G*PR107*+/+^ and HaCaT^*GPR107*-/-^ cells. Data are presented as mean ± SD (*n* = 3), *p < 0.05. **C** Western blot analysis of ubiquitination levels in cell lysates immunoprecipitated with an anti-BECN1 antibody. HaCaT^G*PR107*+/+^, HaCaT^*GPR107*-/-^, and *GPR107*-rescued HaCaT^*GPR107*-/-^ cells were treated with MG132 (100 µM for 6 h) prior to lysis. **D** Western blot analysis of specific BECN1 ubiquitination linkages (HA-Ub) in control keratinocytes versus CUL3 knockdown cells (sh*CUL3*). HaCaT cells were co-transfected with Flag-*BECN1* and the HA-Ub mutants. K6, K11, K27, K29, K33, K48 and K63 indicate that all lysines, except K6, K11, K27, K29, K33, K48 or K63, respectively, were mutated to Arginines. Prior to lysis, cells were treated with MG132 (10 µM) for 6 h. Subsequently, Flag-BECN1 was immunoprecipitated (IP) using an anti-Flag antibody, followed by Western blot analysis using anti-HA and anti-Flag antibodies. **E** MS analysis identified ubiquitinated peptides and the corresponding lysine site (K206) on the BECN1 protein. **F** Co-IP and Western blot analysis of K48-linked BECN1 ubiquitination levels in control keratinocytes versus CUL3 knockdown cells (sh*CUL3*). HaCaT cells were co-transfected with HA-Ub-K48, Flag-*BECN1* (WT) or Flag-*BECN1* (K206R mutant). Prior to lysis, cells were treated with MG132 (10 µM) for 6 h. Subsequently, HA-Ub-K48 was immunoprecipitated (IP) using an anti-HA antibody, and the resulting complexes were detected by Western blot analysis using anti-HA and anti-Flag antibodies.

To determine whether GPR107 regulates CUL3 expression, we detected CUL3 levels in mouse skin tissues and IMQ-induced psoriasiform lesions. As shown in Fig. 5A, CUL3 was expressed at higher levels in normal skin tissues from both *Krt14*^*Cre/+*^*-Gpr107*^*f/f*^ and *Krt14*^*+/+*^*-Gpr107*^*f/f*^ mice. In IMQ-induced psoriasiform lesions, CUL3 expression markedly decreased; however, GPR107 deficiency resulted in higher CUL3 levels in the lesions from IMQ-treated *Krt14*^*Cre/+*^*-Gpr107*^*f/f*^ mice compared to IMQ-treated *Krt14*^*+/+*^*-Gpr107*^*f/f*^ mice. Similarly, in M5-induced cell models, CUL3 was also significantly upregulated upon *GPR107* knockout compared with normal keratinocytes (Fig. 5B).

To investigate GPR107-mediated BECN1 degradation, which requires ubiquitination for UPS-dependent clearance, we assessed BECN1 ubiquitination levels using co-immunoprecipitation with an anti-BECN1 antibody in both *GPR107* knockout and *GPR107*-rescued keratinocytes. In M5 treated keratinocytes, *GPR107* knockout increased ubiquitinated-BECN1 levels when the UPS was inhibited by MG132 prior to protein lysis (Fig. 5C). However, rescuing GPR107 in HaCaT^*GPR107-/-*^ cells under M5 treatment reduced the ubiquitination levels of BECN1. Collectively, these findings indicated that GPR107 negatively regulates CUL3 expression, thereby reducing ubiquitinated-BECN1 and indirectly modulates BECN1 protein levels.

To specify the type of polyubiquitin chains formed on BECN1 modified by CUL3, ubiquitination assays were performed using different ubiquitin mutant vectors (targeting linkages K6, K11, K27, K29, K33, K48, and K63, respectively). In keratinocytes treated with MG132 prior to protein lysis to inhibit the UPS, knockdown of CUL3 failed to alter the K6-, K11-, K27-, K29-, K33-, and K63-linked ubiquitination levels of BECN1, but specifically altered the K48-linked ubiquitination of BECN1 (Fig. 5D). These results indicate that CUL3 primarily mediates K48-linked ubiquitin chain formation on BECN1 protein.

Subsequently, BECN1 was immunoprecipitated using a specific BECN1 antibody, and the resulting ubiquitinated peptides were analyzed by MS. As shown in Fig. 5E, only one ubiquitinated peptide (KIVAENLEK), corresponding to lysine 206, was detected, despite the presence of 29 lysine residues in BECN1 protein. To investigate the functional relevance of this site, we overexpressed Flag-tagged *BECN1* bearing a K206R mutation in keratinocytes. Following sh*CUL3* transduction and subsequent immunoprecipitation assays, the ubiquitination level of the BECN1 K206R mutant remained unchanged upon *CUL3* knockdown, demonstrating that lysine 206 is essential for CUL3-mediated ubiquitination of BECN1 (Fig. 5F). Overall, GPR107 promotes K48-linked ubiquitination of BECN1 at the K206 residue *via* CUL3, thereby potentially facilitating UPS-dependent degradation of BECN1.

### GPR107 promotes secretory autophagy in psoriatic keratinocytes *via* the β-arrestin/ERK/NF-κB/CUL3 axis

**Fig. 6:**
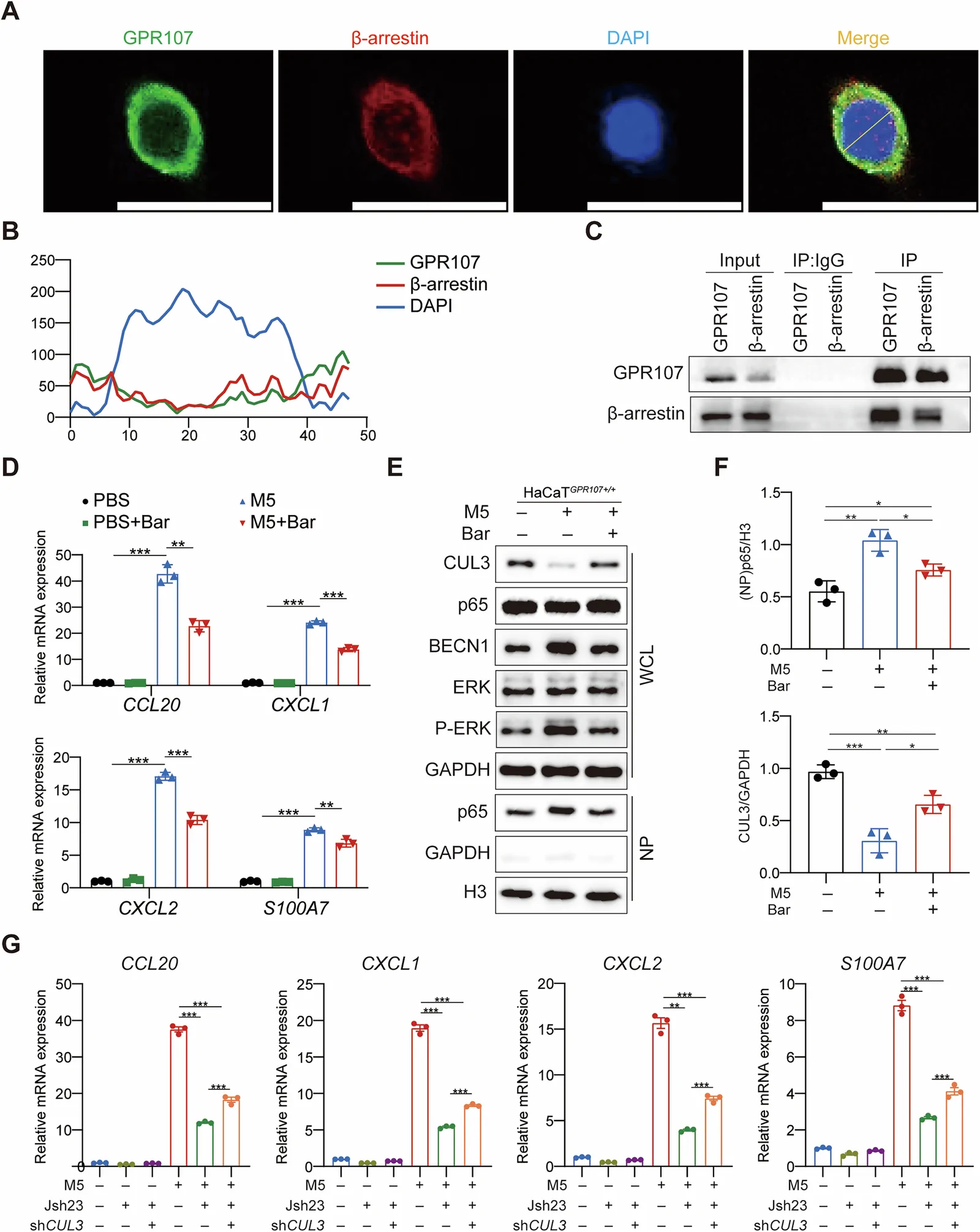
Activation of the β-arrestin/ERK/NF-κB (p65)/CUL3 signal axis by GPR107 increases BECN1 and promotes autophagy-based secretion in psoriatic keratinocytes. **A** Immunofluorescence staining of GPR107 (green) and β-arrestin (red) in HaCaT cells. Nuclei were counterstained with DAPI (blue). Scale bar = 20 µm. **B** The dashed line in the merged image indicates the region of interest for analyzing the distance between GPR107, β-arrestin, and DAPI using ImageJ. **C** Co-immunoprecipitation of GPR107 and β-arrestin in HaCaT cells. Rabbit IgG was used as a negative control. **D** qRT-PCR analysis of *CCL20*, *CXCL1*, *CXCL2*, and *S100A7* mRNA levels in HaCaT cells treated with barbadin (10 µM), followed by M5 treatment for 24 h. Data are presented as mean ± SD (*n* = 3). **p < 0.01, ***p < 0.001. **E** Western blot analysis showing the effect of β-arrestin inhibition on M5-induced activation of the ERK/NF-κB (p65)/CUL3/BECN1 axis. HaCaT cells were treated with the β-arrestin inhibitor barbadin (Bar, 10 µM) for 24 h, followed by M5 stimulation (or no stimulation) for 24 h. **F** Western blot analysis of CUL3 and nuclear p65 protein levels in HaCaT cells with M5 and/or barbadin (Bar, 10 µM). Data are presented as mean ± SD (*n* = 3), *p < 0.05, **p < 0.01, ***p < 0.001. WCL, whole cell lysate; NP, nuclear proteins. **G** mRNA expression levels of chemokines (*CCL20*, *CXCL1*, *CXCL2*) and the antimicrobial peptide *S100A7* in HaCaT cells under various treatment conditions. Treatments included 24 h of M5 stimulation, with or without pre-treatment of the p65 nuclear import inhibitor JSH-23 (20 µM, 24 h) and/or transfection of the sh*CUL3* plasmid. Data are presented as the mean ± SD (*n* = 3). **p < 0.01, ***p < 0.001.

Given that GPR107 primarily regulates its target CUL3, we then sought to identify the downstream signaling pathways activated by GPR107. Firstly, both immunofluorescence and co-immunoprecipitation assays confirmed that β-arrestin interacts with GPR107 (Fig. 6A–C). Furthermore,the β-arrestin inhibitor barbadin led to a significant reduction in the M5-induced high levels of chemokines (*CCL20*, *CXCL1*, *CXCL2*) and the antimicrobial peptide *S100A7* (Fig. 6D). These results indicate that GPR107 mediates signaling in a β-arrestin-dependent manner, which may contribute to secretory autophagy in psoriatic keratinocytes.

Then, RNA-seq analysis of M5-stimulated *GPR107*-knockout keratinocytes showed substantial alterations in gene expression profiles following *GPR107* deletion (Fig. S5B). Pathway analysis further showed that M5 stimulation influenced autophagy-related processes and NF-κB signaling in *GPR107*-deficient keratinocytes compared to control cells (Fig. S5C). As, the ERK pathway is commonly activated by β-arrestin-mediated signaling, we investigated the potential involvement of the ERK/NF-κB (p65) pathway in regulating *CUL3* and *BECN1* expression. As expected, M5 stimulation increased phosphorylated ERK (p-ERK) and nuclear p65 levels in keratinocytes, leading to *CUL3* downregulation and *BECN1* upregulation (Fig. 6E). These M5-induced alterations were entirely reversed by barbadin. Furthermore, the resulting *CUL3* downregulation depended on the nuclear translocation of p65 triggered by M5 stimulation (Fig. 6F, S6A, B).

Since the canonical NF-κB pathway controls the transcription of psoriasis-related chemokines and antimicrobial peptides, we further investigated whether GPR107 mediates autophagy-based secretion *via* the β-arrestin/ERK/NF-κB/CUL3 signaling axis. As shown in Fig. 6G, the p65 nuclear import inhibitor JSH-23 significantly decreased the M5-stimulated upregulation of chemokines and *S100A7*. Notably, this inhibitory effect was markedly reversed upon *CUL3* knockdown. Strikingly, CUL3 overexpression substantially reduced the levels of chemokines and antimicrobial peptides, even in the presence of robust upstream NF-κB activation (Fig. S6C and S6D). Overall, these results indicate that GPR107 regulates the release of chemokines and antimicrobial peptides through a coordinated dual mechanism: it not only initiates canonical NF-κB-driven transcription but also essentially couples it with NF-κB/CUL3-dependent secretory autophagy, thereby collectively regulating the secretion of chemokines and antimicrobial peptides.

### Downregulation of GPR107 ameliorates phenotypes in IMQ-induced psoriasiform dermatitis

**Fig. 7:**
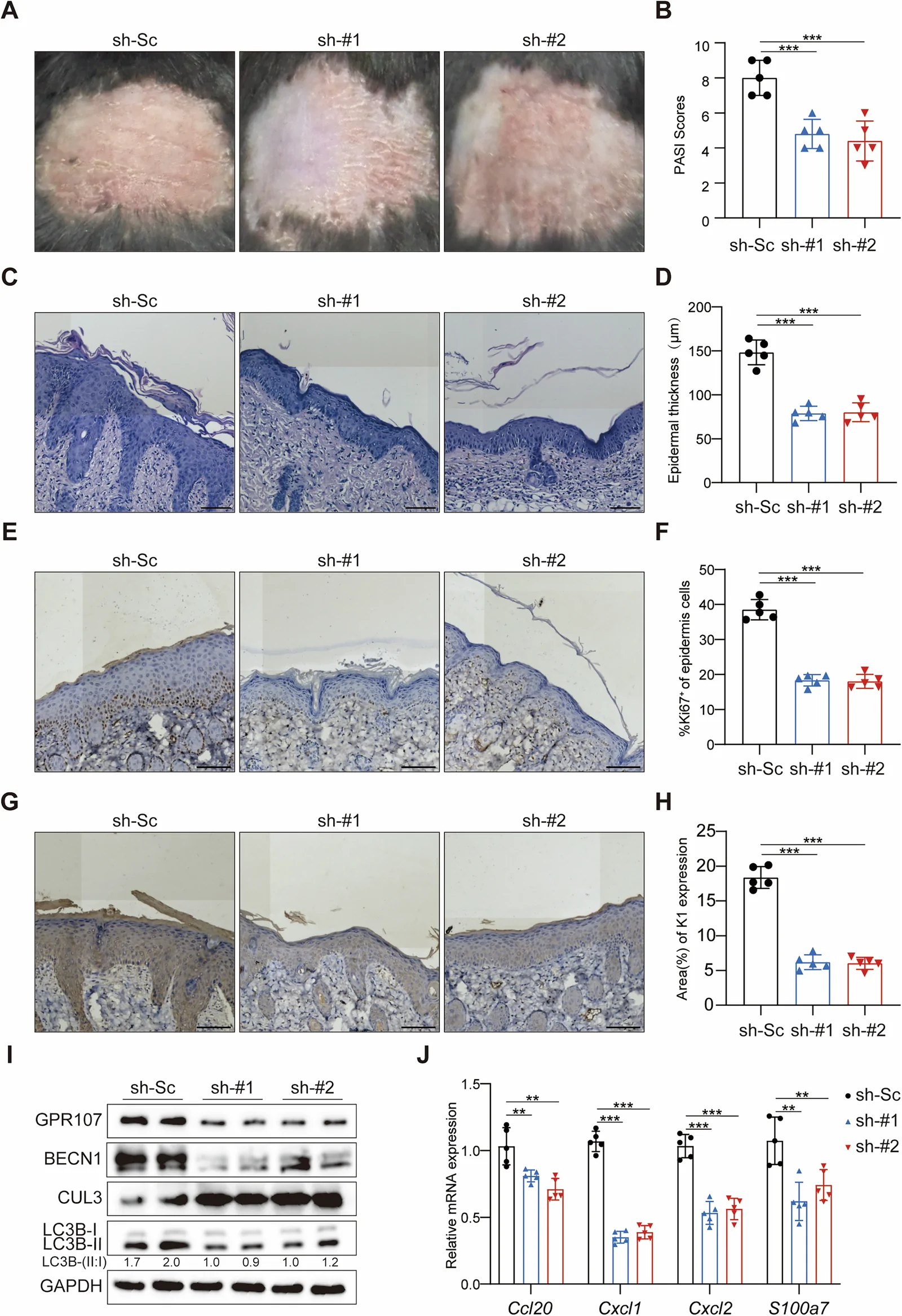
*Gpr107* knockdown ameliorates IMQ-induced psoriasiform phenotypes. **A** Representative images of 6-day IMQ-induced psoriasiform lesions from mice with *Gpr107* knockdown. On each day, viral supernatants (5.0–6.0 × 10^7^ transducing units) containing Scramble shRNA (sh-Sc), shRNA-*Gpr107*#1 (sh-#1), or shRNA-*Gpr107*#2 (sh-#2) was smeared to dorsal lesions immediately after a 6-h IMQ application. **B** Analysis of PASI scores in 6-day IMQ-induced psoriasiform lesions. Data are presented as mean ± SD (n = 5), ***p < 0.001. **C** Representative H&E-stained histological sections of dorsal skin from IMQ-treated mice. Mice received viral supernatants containing Scramble shRNA (sh-Sc) or *Gpr107* knockdown constructs (sh-#1 and sh-#2). Scale bars = 100 μm. **D** Quantitative analysis of epidermal thickness in the dorsal skin sections shown in (**C**). Data are presented as mean ± SD (*n* = 5). ***p < 0.001. **E**, **F** Immunohistochemistry and quantitative analysis of Ki-67-positive cells in 6-day IMQ-induced psoriasiform lesions from mice treated with viral supernatants containing Scramble shRNA (sh-Sc) or *Gpr107* knockdown constructs (sh-#1 and sh-#2). Scale bars = 100 μm. Data are presented as mean ± SD (*n* = 5). ***p < 0.001. **G**, **H** Immunohistochemistry and quantitative analysis of keratin 1 (K1) expression in 6-day IMQ-induced psoriasiform lesions from mice treated with viral supernatants containing Scramble shRNA (sh-Sc) or *Gpr107* knockdown constructs (sh-#1 and sh-#2). Scale bars = 100 μm. Data are presented as mean ± SD (*n* = 5). ***p < 0.001. **I** Western blot analysis of GPR107, BECN1, CUL3, and LC3B-II/LC3B-I in skin tissue lysates from 6-day IMQ-induced psoriasiform lesions in mice treated with viral supernatants containing Scramble shRNA (sh-Sc) or *Gpr107* knockdown constructs (sh-#1 and sh-#2). **J** qRT-PCR analysis of *Ccl20*, *Cxcl1*, *Cxcl2*, and *S100a7* mRNA levels in skin lysates from 6-day IMQ-induced psoriasiform lesions of mice treated with viral supernatants containing Scramble shRNA (sh-Sc) or *Gpr107* knockdown constructs (sh-#1 and sh-#2). Data are presented as mean ± SD (*n* = 5). **p < 0.01, ***p < 0.001.

Based on evidence that GPR107-mediated signaling through the β-arrestin/ERK/NF-κB/CUL3 axis regulates secretory autophagy in psoriatic keratinocytes, we attempted to attenuate IMQ-induced psoriasiform lesions by knocking down *Gpr107* expression. As show in Fig. 7A, a marked attenuation of scaling and erythema was observed in IMQ-induced psoriasiform lesions following the application of shRNA-*Gpr107*. In vivo knockdown of *Gpr107* resulted in a significant decrease in the PASI score compared with controls (Fig. 7B). Histological analysis on Day 6 showed that *Gpr107* shRNA treatment led to a significant decrease in mouse back skin thickness compared to the scrambled shRNA control (Fig. 7C, D). Immunohistochemistry analysis revealed two key changes in *Gpr107* shRNA-treated mice compared to controls: a significant decrease in the ratio of Ki-67 positive keratinocytes in IMQ-induced psoriasiform lesions (Fig. 7E, F and S7A), and a significant reduction in keratin 1 expression (Fig. 7G, H and S7B).

Furthermore, in vivo knockdown of *Gpr107* subsequently led to reciprocal changes in autophagy regulators within IMQ-induced psoriasiform lesions: specifically, CUL3 expression was increased while BECN1 expression was reduced. Correspondingly, the LC3B-II/LC3B-I ratio was markedly decreased (Fig. 7I). We next examined the impact of *Gpr107* knockdown on cellular secretory autophagy by measuring levels of the chemokines *Ccl20*, *Cxcl1*, and *Cxcl2*, as well as the antimicrobial peptide *S100a7*. Knockdown using *Gpr107* shRNA resulted in a reduction of these chemokines and the antimicrobial peptide across both treatment groups (Fig. 7J). In summary, *Gpr107* knockdown is associated with reduced phenotypic severity of IMQ-induced psoriasiform lesions, potentially by suppressing GPR107-mediated secretory autophagy in keratinocytes.

## Discussion

In this study, we demonstrated that GPR107 functions as a key upstream regulator mediating both cell proliferation and BECN1-dependent secretory autophagy in psoriatic keratinocytes. Mechanistically, by activating the β-arrestin/ERK/NF-κB pathway, GPR107 regulates CUL3 expression, thereby modulating K48-linked ubiquitination and proteasomal degradation of BECN1. Our findings provided a highlighted novel function of GPR107 in inflammatory diseases.

GPR107 depends on the CME pathway for its role in the endocytic trafficking of cargos such as collagen IV and transferrin [29–31]. Internalized GPR107 interacts with clathrin and the retromer component VPS35 [29, 35], suggesting that GPR107 is likely to mediate signal transduction processes regulating the recycling of receptors from endocytic compartments to the plasma membrane. In the present study, we confirmed that GPR107 upregulation not only promotes the proliferation of psoriatic keratinocytes both in vitro and in vivo, but also modulates secretion of keratinocyte-derived chemokines (including CCL20, CXCL1, and CXCL2) and the antimicrobial peptide S100A7. Interestingly, keratinocyte-specific *Gpr107* knockout led to markedly decreased levels of autophagy, consistent with attenuated psoriatic phenotypes, including reduced cutaneous inflammation in IMQ-induced psoriasiform lesions, a lower keratinocyte proliferation rate, and diminished secretion of keratinocyte-derived chemokines. Therefore, GPR107-mediated endocytosis and its associated signaling contribute to the regulation of secretory autophagy in psoriatic keratinocytes.

BECN1-dependent autophagy is a hallmark of BECN1 function, as BECN1 serves as a platform protein that assembles an interactome of diverse partners regulating the initiation of autophagosome formation and distinct stages of endocytosis [19, 20]. It is well established that BECN1 interacts with Vps34 to form three functionally distinct complexes: one with ATG14L that initiates autophagosome formation, one with UVRAG that promotes autophagosome maturation and endocytic trafficking, and one with UVRAG-RUBICON, in which RUBICON inhibits these processes by suppressing Vps34 activity [19]. Interestingly, GPR107 upregulation is associated with increased levels of both autophagy and BECN1 protein in psoriatic keratinocytes. Conversely, GPR107 deficiency significantly reduces BECN1 protein levels and inhibits autophagy in psoriatic keratinocytes. These alterations in BECN1 do not result in changes in the levels of BECN1-bound BCL2, ATG14L, UVRAG, or RUBICON. Owing to the binding of BCL2 to BECN1, increased cell proliferation persisted in psoriatic keratinocytes. Notably, we found that psoriatic keratinocytes exhibit alterations in BECN1 protein rather than its mRNA, suggesting that GPR107-mediated modulation of BECN1-dependent secretory autophagy is linked to proteasome-mediated degradation of BECN1.

K48-linked, ubiquitin-dependent targeting of proteins is a hallmark of proteasome-mediated degradation in mammalian cells [36]. Indeed, accumulating evidence suggests that the stability of the inhibitory BECN1 complex is regulated by post-translational modifications of the BECN1 protein induced by upstream signaling pathways [19, 37]. In this study, we identified BECN1-bound CUL3 as an E3 ubiquitin ligase involved in BECN1 ubiquitination. CUL3 mediates K48-linked ubiquitination at lysine 206 (K206) of BECN1. Specifically, CUL3 expression levels are negatively regulated by GPR107 in psoriatic keratinocytes, which is consistent with the observed K48-linked ubiquitination of BECN1 and the corresponding changes in BECN1 protein levels.

It is now widely acknowledged that receptor internalization and trafficking constitute key mechanisms underlying GPCR signaling [38–42]. Specifically, intracellular signaling can be initiated in an internalization-dependent manner. β-arrestin mediates clathrin-dependent GPCR endocytosis and regulates ERK1/2 signaling through scaffolding interactions within the CME machinery [38]. In this study, we demonstrated that internalized GPR107 mediates β-arrestin-dependent signal transduction, leading to ERK1/2 activation and NF-κB nuclear translocation. The activated nuclear p65 subunit, in turn, negatively regulates CUL3 expression, thereby reducing K48-linked ubiquitination of BECN1 and enhancing BECN1-dependent secretory autophagy in psoriatic keratinocytes.

In summary, internalized GPR107 negatively regulates CUL3 expression, thereby reducing K48-linked ubiquitination and proteasomal degradation of BECN1. This stabilization of BECN1 facilitates secretory autophagy in psoriatic keratinocytes and promotes their proliferation and inflammatory responses. GPR107 targeting of CUL3 depends on activation of the β-arrestin/ERK/NF-κB pathway. Although no inhibitor of GPR107 has been identified, autophagy-based secretion is markedly reduced in psoriatic keratinocytes when the β-arrestin/ERK/NF-κB/CUL3/BECN1 axis is inhibited or blocked, highlighting potential new therapeutic targets for psoriasis.

## Supplementary information

Supplementary Data

Supplementary Figures 1

Supplementary Figures 2

Supplementary Figures 3

Supplementary Figures 4

Supplementary Figures 5

Supplementary Figures 6

Supplementary Figures 7

Supplementary Original western blots

## Data Availability

The data that support the findings of this study are available upon request from the corresponding author.

## References

[1] Svedbom A, Mallbris L, Larsson P, Nikamo P, Wolk K, Kjellman P, et al. Long-term outcomes and prognosis in new-onset psoriasis. JAMA Dermatol. 2021;157:1–8. 33851956 10.1001/jamadermatol.2021.0734 PMC8047767

[2] Greb JE, Goldminz AM, Elder JT, Lebwohl MG, Gladman DD, Wu JJ, et al. Psoriasis. Nat Rev Dis Primers. 2016;2:16082. 27883001 10.1038/nrdp.2016.82

[3] Boehncke WH, Schön MP. Psoriasis. Lancet. 2015;386:983–94. 26025581 10.1016/S0140-6736(14)61909-7

[4] Griffiths CE, Barker JN. Pathogenesis and clinical features of psoriasis. Lancet. 2007;370:263–71. 17658397 10.1016/S0140-6736(07)61128-3

[5] Kim J, Krueger JG. The immunopathogenesis of psoriasis. Dermatol Clin. 2015;33:13–23. 25412780 10.1016/j.det.2014.09.002

[6] Luo Y, Pang B, Hao J, Li Q, Qiao P, Zhang C, et al. Keratin 17 covalently binds to alpha-enolase and exacerbates proliferation of keratinocytes in psoriasis. Int J Biol Sci. 2023;19:3395–411. 37497003 10.7150/ijbs.83141 PMC10367554

[7] Ma F, Plazyo O, Billi AC, Tsoi LC, Xing X, Wasikowski R, et al. Single cell and spatial sequencing define processes by which keratinocytes and fibroblasts amplify inflammatory responses in psoriasis. Nat Commun. 2023;14:3455. 37308489 10.1038/s41467-023-39020-4 PMC10261041

[8] Zhou X, Chen Y, Cui L, Shi Y, Guo C. Advances in the pathogenesis of psoriasis: from keratinocyte perspective. Cell Death Dis. 2022;13:81. 35075118 10.1038/s41419-022-04523-3 PMC8786887

[9] Nestle FO, Di Meglio P, Qin JZ, Nickoloff BJ. Skin immune sentinels in health and disease. Nat Rev Immunol. 2009;9:679–91. 19763149 10.1038/nri2622 PMC2947825

[10] Quaresma JAS. Organization of the skin immune system and compartmentalized immune responses in infectious diseases. Clin Microbiol Rev. 2019;32:10–1128. 10.1128/CMR.00034-18 PMC6750136 31366611

[11] Hao J, Yu J, Yorek MS, Yu CL, Pope RM, Chimenti MS, et al. Keratinocyte FABP5-VCP complex mediates recruitment of neutrophils in psoriasis. Cell Rep. 2023;42:113449. 37967009 10.1016/j.celrep.2023.113449 PMC10729729

[12] Tu Z, Zhang S, Zhou G, Zhou L, Xiang Q, Chen Q, et al. LMO4 is a disease-provocative transcription coregulator activated by IL-23 in psoriatic keratinocytes. J Investig Dermatol. 2018;138:1078–87. 29258893 10.1016/j.jid.2017.12.010

[13] Long F, Wei X, Chen Y, Li M, Lian N, Yu S, et al. Gasdermin E promotes translocation of p65 and c-jun into nucleus in keratinocytes for progression of psoriatic skin inflammation. Cell Death Dis. 2024;15:180. 38429278 10.1038/s41419-024-06545-5 PMC10907691

[14] Kim KH, Lee MS. Autophagy-a key player in cellular and body metabolism. Nat Rev Endocrinol. 2014;10:322–37. 24663220 10.1038/nrendo.2014.35

[15] Li W, He P, Huang Y, Li YF, Lu J, Li M, et al. Selective autophagy of intracellular organelles: recent research advances. Theranostics. 2021;11:222–56. 33391472 10.7150/thno.49860 PMC7681076

[16] Clarke AJ, Simon AK. Autophagy in the renewal, differentiation and homeostasis of immune cells. Nat Rev Immunol. 2019;19:170–83. 30531943 10.1038/s41577-018-0095-2

[17] Shibutani ST, Saitoh T, Nowag H, Münz C, Yoshimori T. Autophagy and autophagy-related proteins in the immune system. Nat Immunol. 2015;16:1014–24. 26382870 10.1038/ni.3273

[18] Yin H, Wu H, Chen Y, Zhang J, Zheng M, Chen G, et al. The therapeutic and pathogenic role of autophagy in autoimmune diseases. Front Immunol. 2018;9:1512. 30108582 10.3389/fimmu.2018.01512 PMC6080611

[19] Salminen A, Kaarniranta K, Kauppinen A. Beclin 1 interactome controls the crosstalk between apoptosis, autophagy and inflammasome activation: impact on the aging process. Ageing Res Rev. 2013;12:520–34. 23220384 10.1016/j.arr.2012.11.004

[20] Joubert PE, Meiffren G, Grégoire IP, Pontini G, Richetta C, Flacher M, et al. Autophagy induction by the pathogen receptor CD46. Cell Host Microbe. 2009;6:354–66. 19837375 10.1016/j.chom.2009.09.006

[21] Deretic V, Jiang S, Dupont N. Autophagy intersections with conventional and unconventional secretion in tissue development, remodeling and inflammation. Trends Cell Biol. 2012;22:397–406. 22677446 10.1016/j.tcb.2012.04.008 PMC3408825

[22] Keulers TG, Schaaf MB, Rouschop KM. Autophagy-dependent secretion: contribution to tumor progression. Front Oncol. 2016;6:251. 27933272 10.3389/fonc.2016.00251 PMC5122571

[23] Yang Z, Goronzy JJ, Weyand CM. Autophagy in autoimmune disease. J Mol Med. 2015;93:707–17. 26054920 10.1007/s00109-015-1297-8 PMC4486076

[24] Varshney P, Saini N. PI3K/AKT/mTOR activation and autophagy inhibition plays a key role in increased cholesterol during IL-17A mediated inflammatory response in psoriasis. Biochim Biophys Acta Mol Basis Dis. 2018;1864:1795–803. 29432814 10.1016/j.bbadis.2018.02.003

[25] Buerger C. Epidermal mTORC1 signaling contributes to the pathogenesis of psoriasis and could serve as a therapeutic target. Front Immunol. 2018;9:2786. 30555471 10.3389/fimmu.2018.02786 PMC6284005

[26] Wang Z, Zhou H, Zheng H, Zhou X, Shen G, Teng X, et al. Autophagy-based unconventional secretion of HMGB1 by keratinocytes plays a pivotal role in psoriatic skin inflammation. Autophagy. 2021;17:529–52. 32019420 10.1080/15548627.2020.1725381 PMC8007160

[27] Edgar AJ. Human GPR107 and murine Gpr108 are members of the LUSTR family of proteins found in both plants and animals, having similar topology to G-protein coupled receptors. DNA Seq. 2007;18:235–41. 17454009 10.1080/10425170701207182

[28] Calderón-Zamora L, Canizalez-Román A, León-Sicairos N, Aguilera-Mendez A, Huang F, Hong E, et al. Changes in expression of orphan receptors GPR99 and GPR107 during the development and establishment of hypertension in spontaneously hypertensive rats. J Recept Signal Transduct Res. 2021;41:558–65. 33121311 10.1080/10799893.2020.1835959

[29] Zhou GL, Na SY, Niedra R, Seed B. Deficits in receptor-mediated endocytosis and recycling in cells from mice with Gpr107 locus disruption. J Cell Sci. 2014;127:3916–27. 24849652 10.1242/jcs.135269

[30] Xu D, Tong Z, Yang P, Chen Q, Wang S, Zhao W, et al. G protein-coupled receptor 107 deficiency promotes development of diabetic nephropathy. Mol Biomed. 2025;6:10. 39932642 10.1186/s43556-025-00250-1 PMC11814420

[31] Xu R, Liang J, Zhang S, Moru P, Liao K, Xu D, et al. GPR107: a key driver of breast cancer invasion and metastasis through collagen IV modulation. Cancer Gene Ther. 2025;32:1414–27. 41073571 10.1038/s41417-025-00977-7

[32] Birgisdottir ÅB, Johansen T. Autophagy and endocytosis - interconnections and interdependencies. J Cell Sci. 2020;133:jcs228114. 10.1242/jcs.228114 32501285

[33] Zhao W, Liao K, Song W, Wang J, Cai C, Zhou F, et al. Chaperone-mediated autophagy dysfunction in imiquimod-induced psoriasiform dermatitis. Autophagy Rep. 2025;4:2544061. 40873769 10.1080/27694127.2025.2544061 PMC12380211

[34] Choi MR, Kim HD, Cho S, Jeon SH, Kim DH, Wee J, et al. Anoctamin1 induces hyperproliferation of HaCaT keratinocytes and triggers imiquimod-induced psoriasis-like skin injury in mice. Int J Mol Sci. 2021;22:7145. 10.3390/ijms22137145 PMC8268182 34281197

[35] Tafesse FG, Guimaraes CP, Maruyama T, Carette JE, Lory S, Brummelkamp TR, et al. GPR107, a G-protein-coupled receptor essential for intoxication by Pseudomonas aeruginosa exotoxin A, localizes to the Golgi and is cleaved by furin. J Biol Chem. 2014;289:24005–18. 25031321 10.1074/jbc.M114.589275 PMC4148833

[36] Chau V, Tobias JW, Bachmair A, Marriott D, Ecker DJ, Gonda DK, et al. A multiubiquitin chain is confined to specific lysine in a targeted short-lived protein. Science. 1989;243:1576–83. 2538923 10.1126/science.2538923

[37] Wei Y, Pattingre S, Sinha S, Bassik M, Levine B. JNK1-mediated phosphorylation of Bcl-2 regulates starvation-induced autophagy. Mol Cell. 2008;30:678–88. 18570871 10.1016/j.molcel.2008.06.001 PMC2478643

[38] Wang W, Qiao Y, Li Z. New insights into modes of GPCR activation. Trends Pharmacol Sci. 2018;39:367–86. 29395118 10.1016/j.tips.2018.01.001

[39] Fan L, Wang S. Biased GPCR signaling: possible mechanisms and therapeutic applications. Biochemistry. 2025;64:1180–92. 40016120 10.1021/acs.biochem.4c00827

[40] Chan HCS, Li Y, Dahoun T, Vogel H, Yuan S. New binding sites, new opportunities for GPCR drug discovery. Trends Biochem Sci. 2019;44:312–30. 30612897 10.1016/j.tibs.2018.11.011

[41] Latorraca NR, Wang JK, Bauer B, Townshend RJL, Hollingsworth SA, Olivieri JE, et al. Molecular mechanism of GPCR-mediated arrestin activation. Nature. 2018;557:452–6. 29720655 10.1038/s41586-018-0077-3 PMC6294333

[42] Ahn S, Kaipparettu BA. G-protein coupled receptors in metabolic reprogramming and cancer. Pharmacol Ther. 2025;270:108849. 40204142 10.1016/j.pharmthera.2025.108849 PMC13108698

