# Supplementary material for: GPR107 promotes autophagy secretion in psoriatic keratinocytes by inhibiting BECN1 K48-linked ubiquitination
Source: Cell Death Dis. 2026 Jun 26;17(1):796. doi: 10.1038/s41419-026-09038-9 (PMC13572499; doi:10.1038/s41419-026-09038-9)

Fig1F

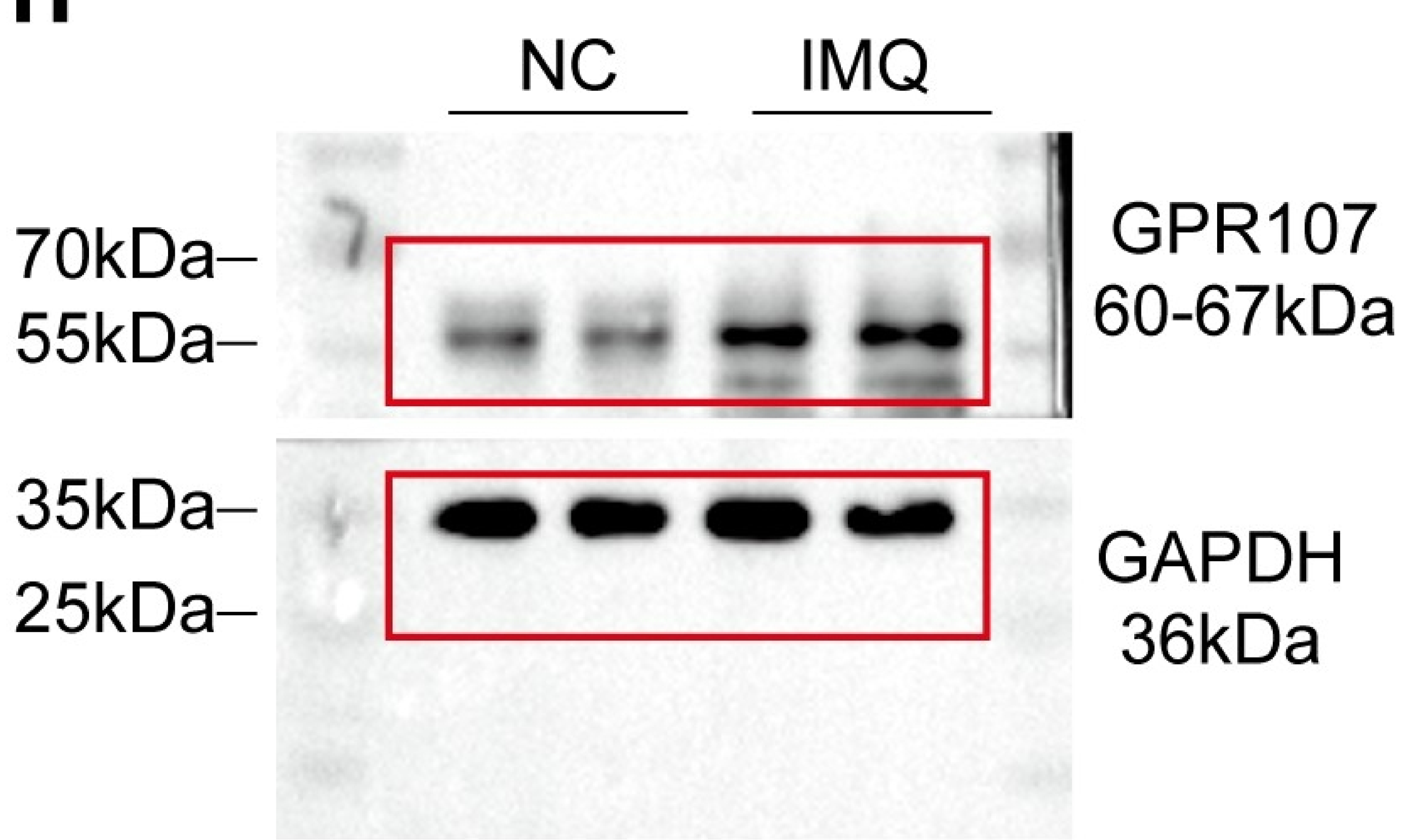

Fig1I

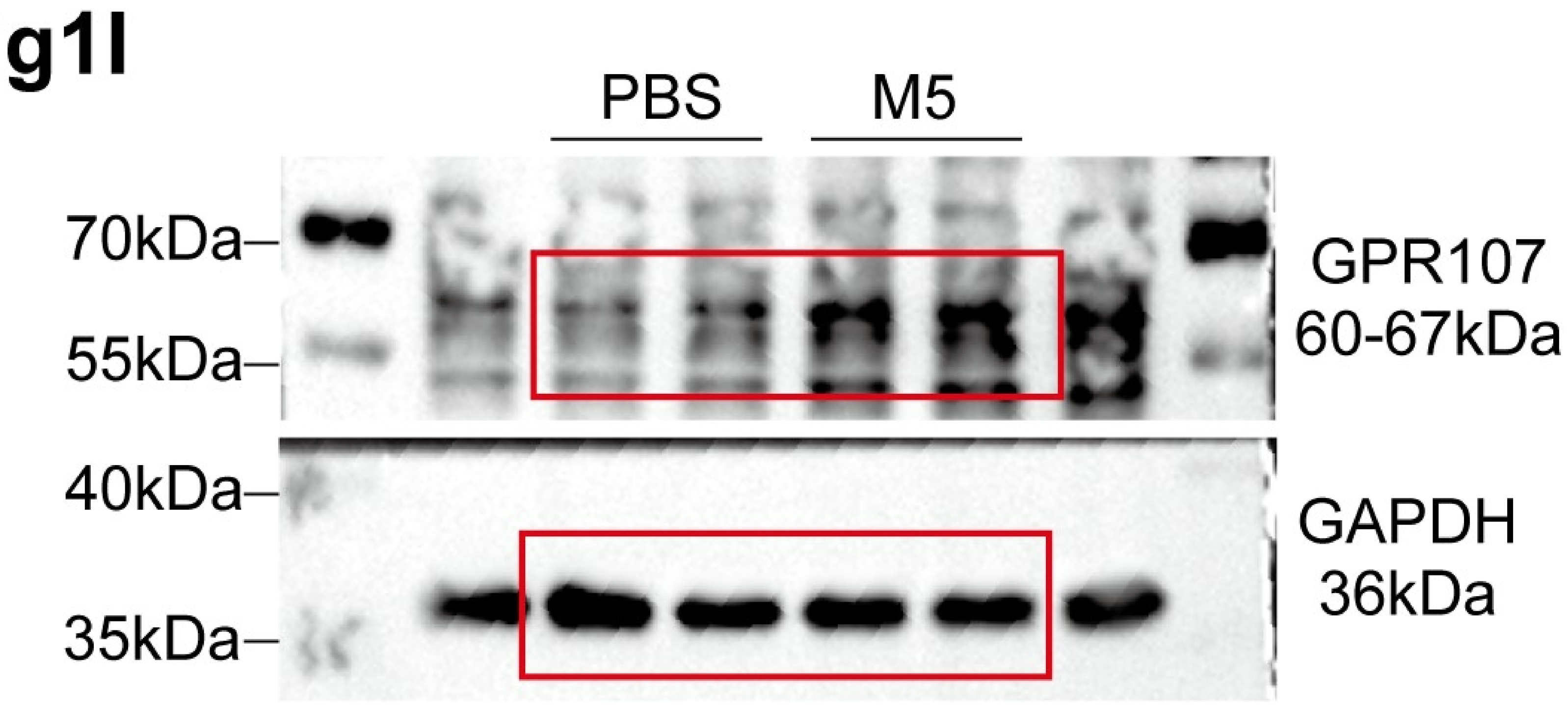

Fig3C

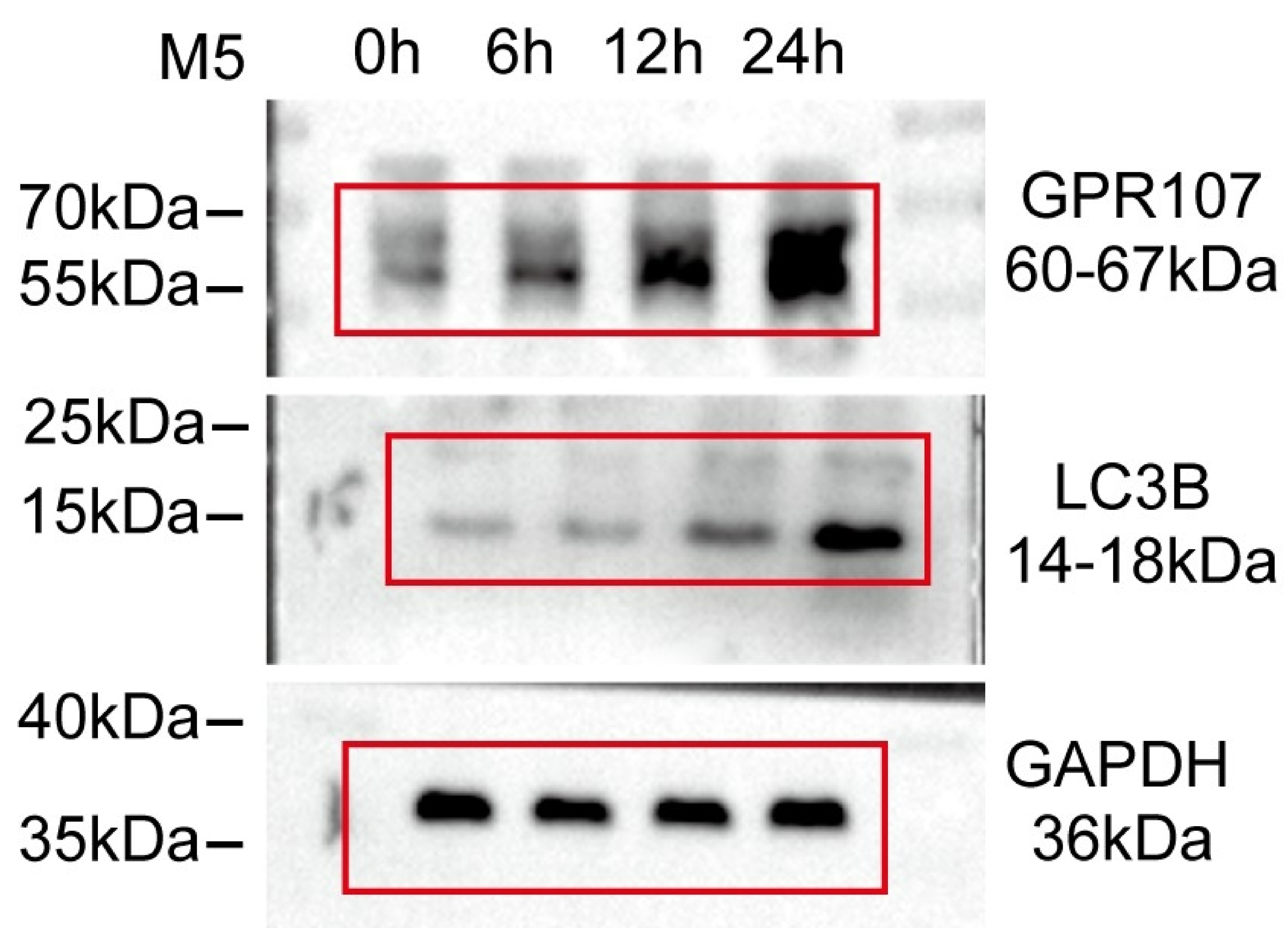

Fig3H

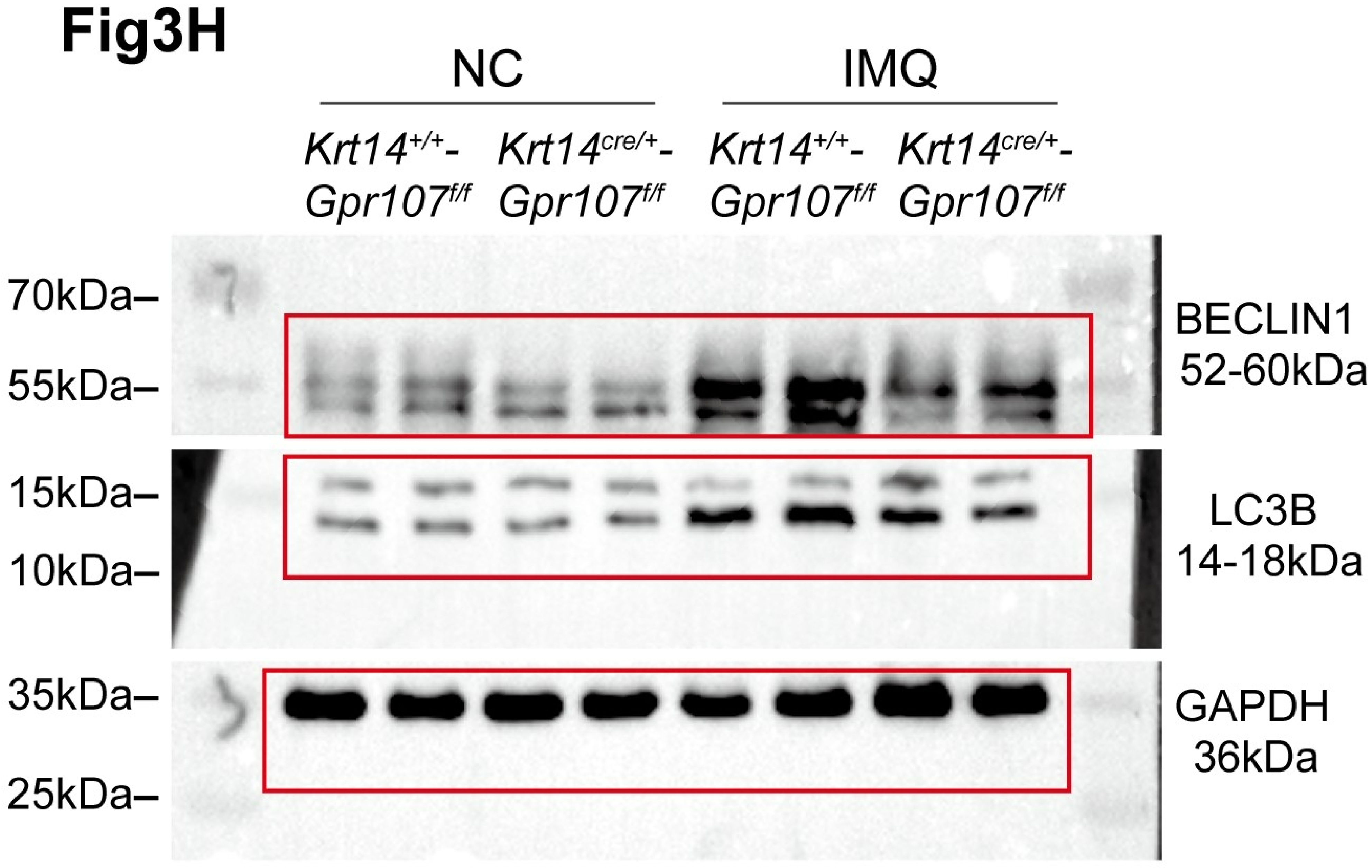

Fig3I

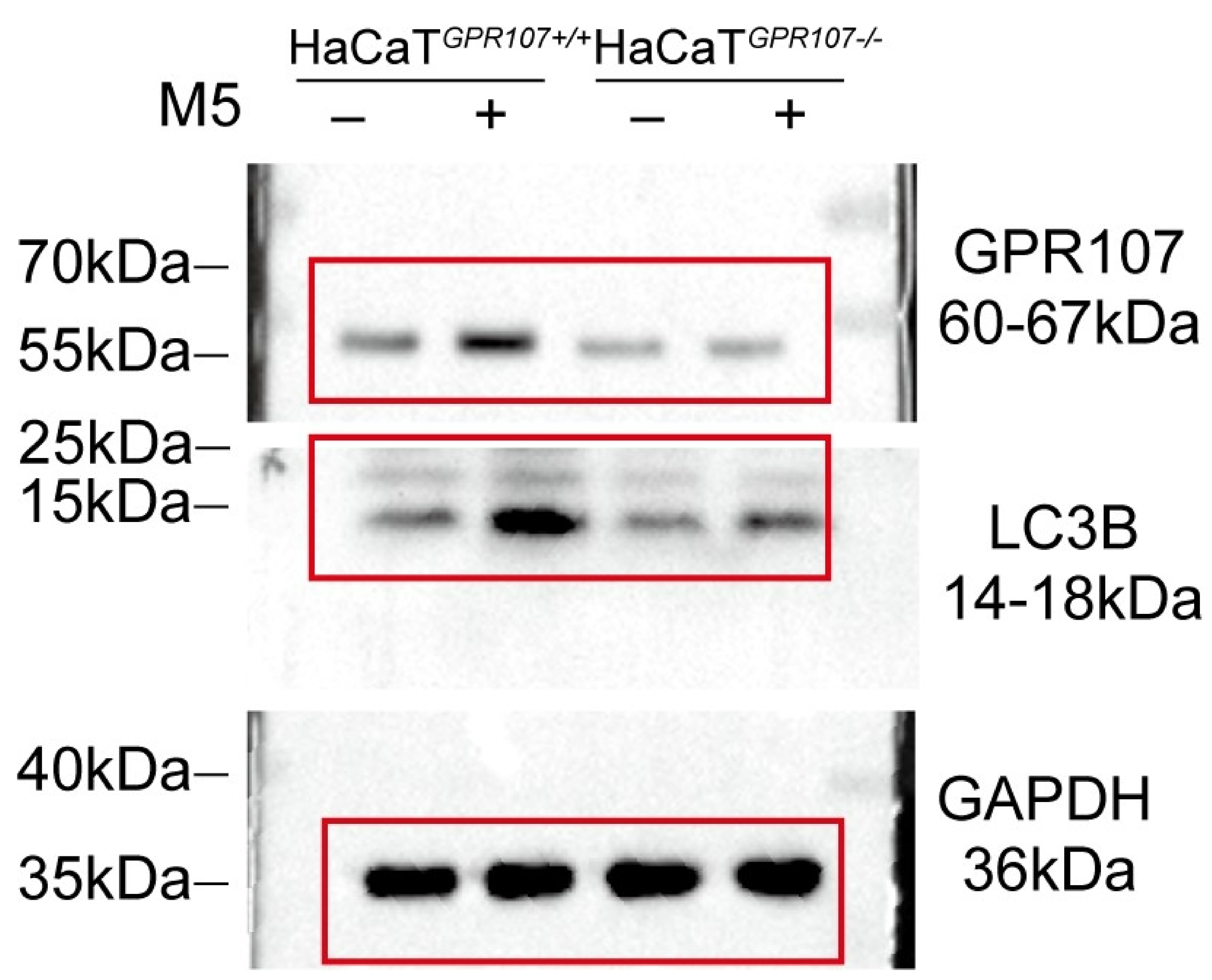

Fig3J

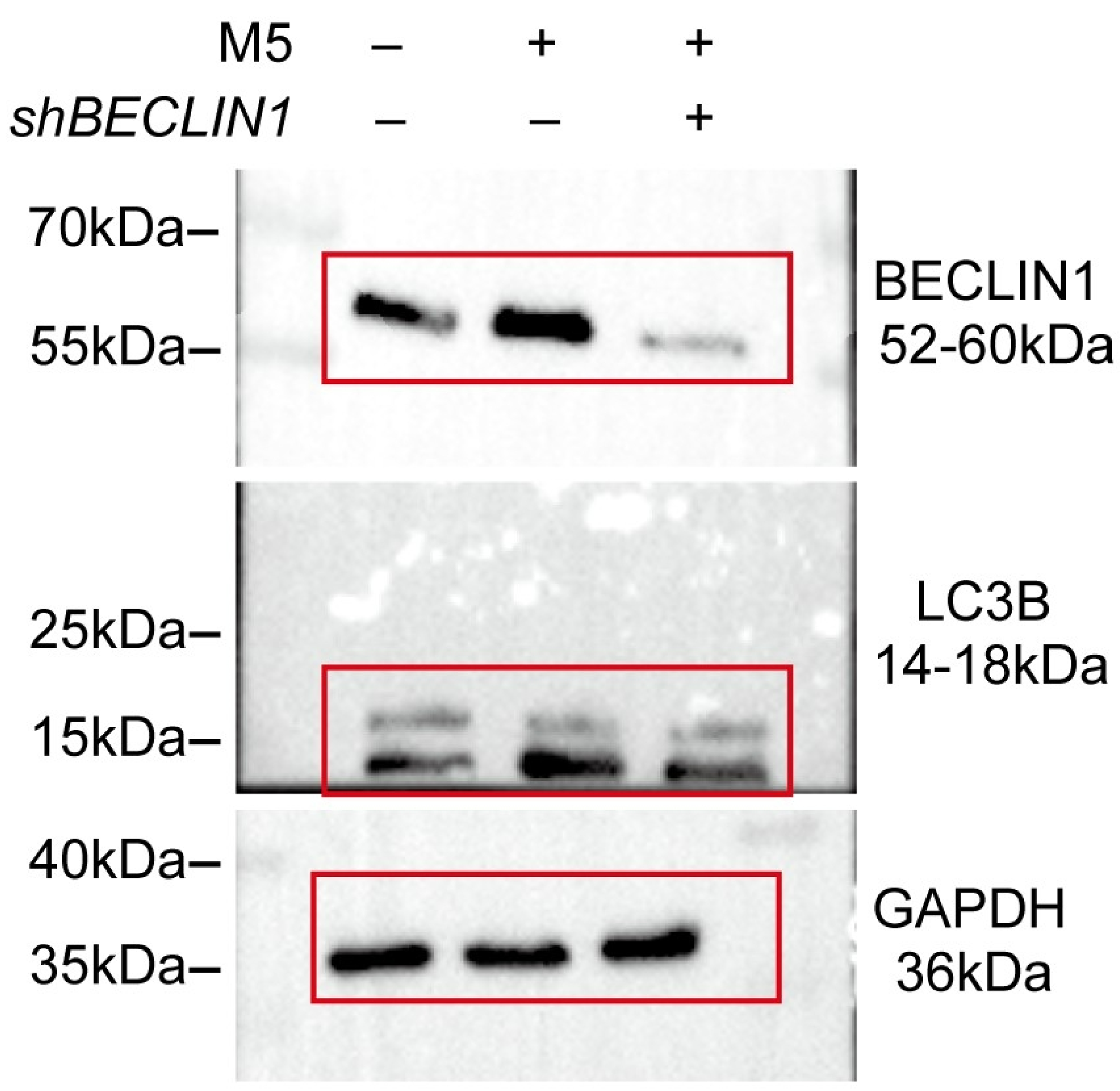

Fig4B

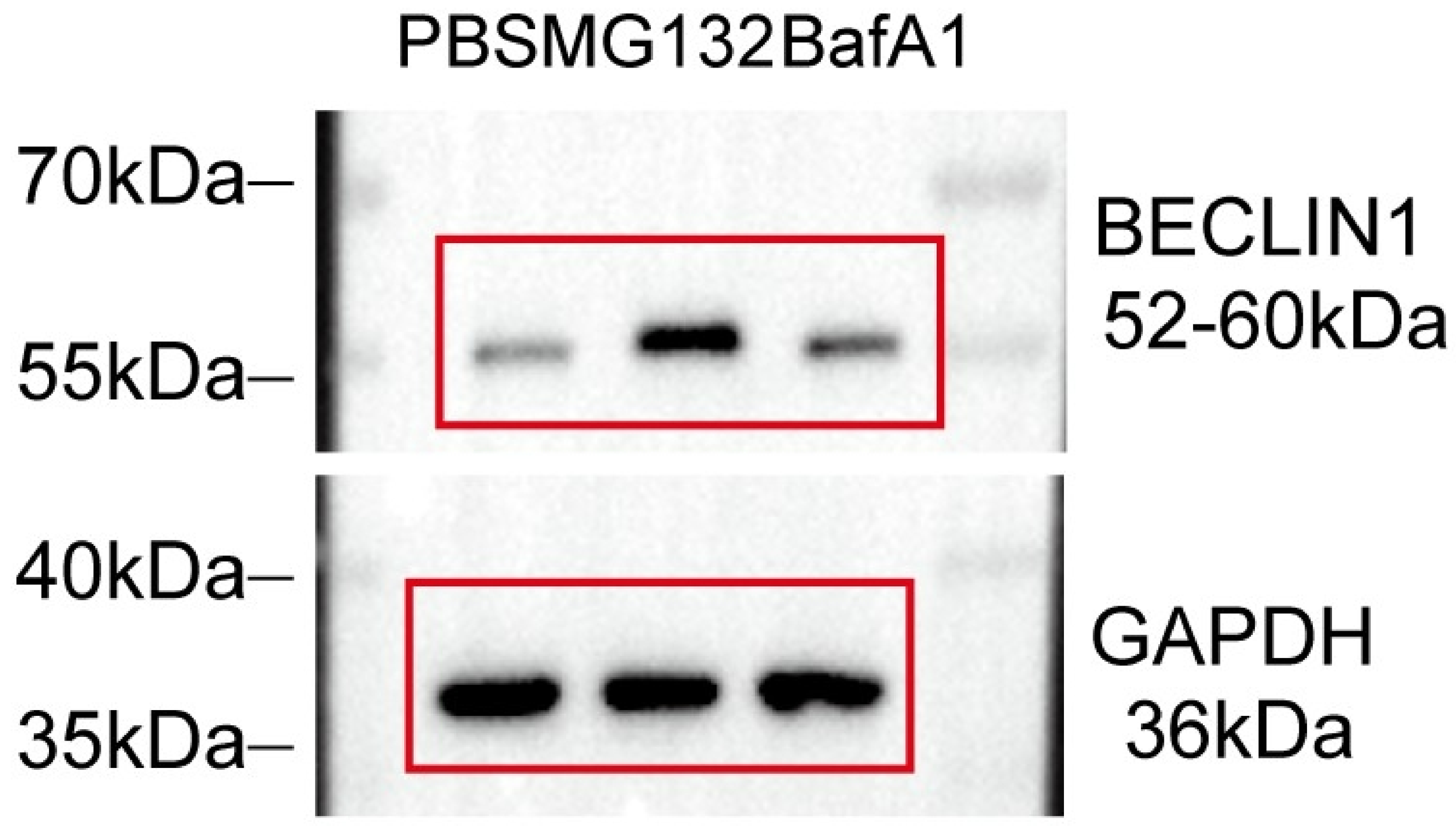

Fig4C

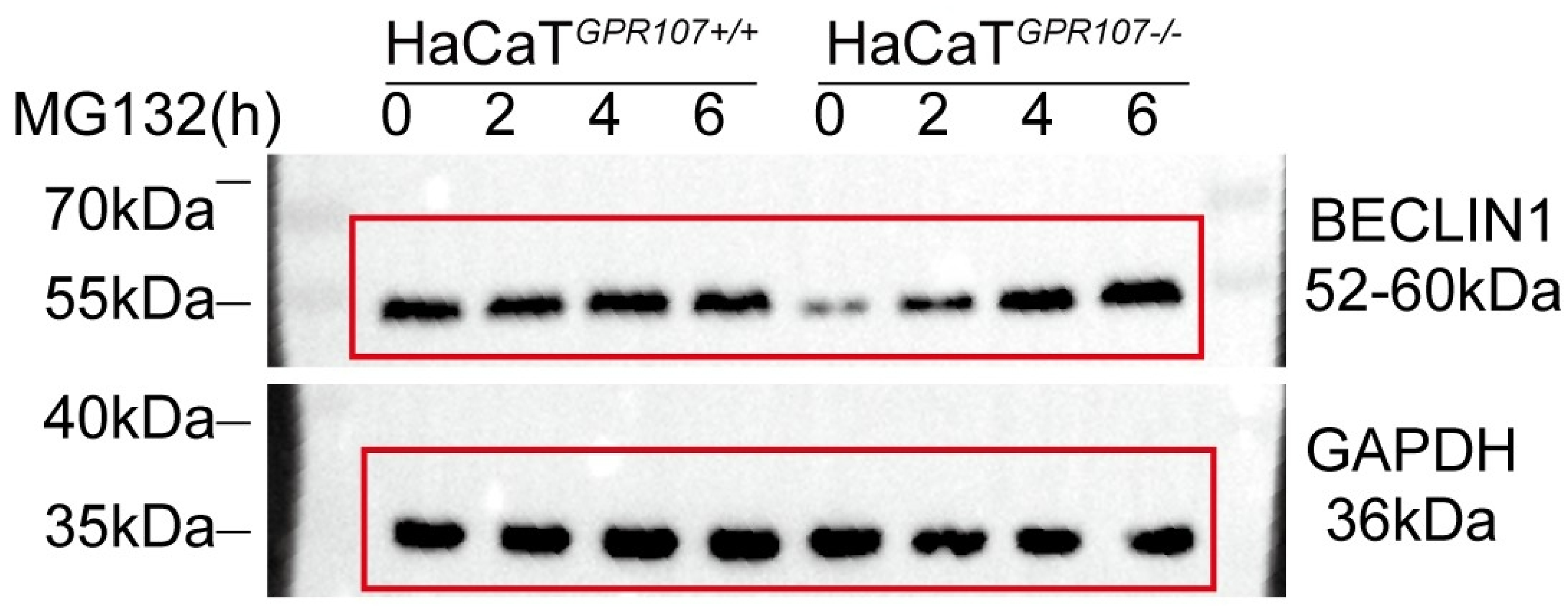

Fig4D

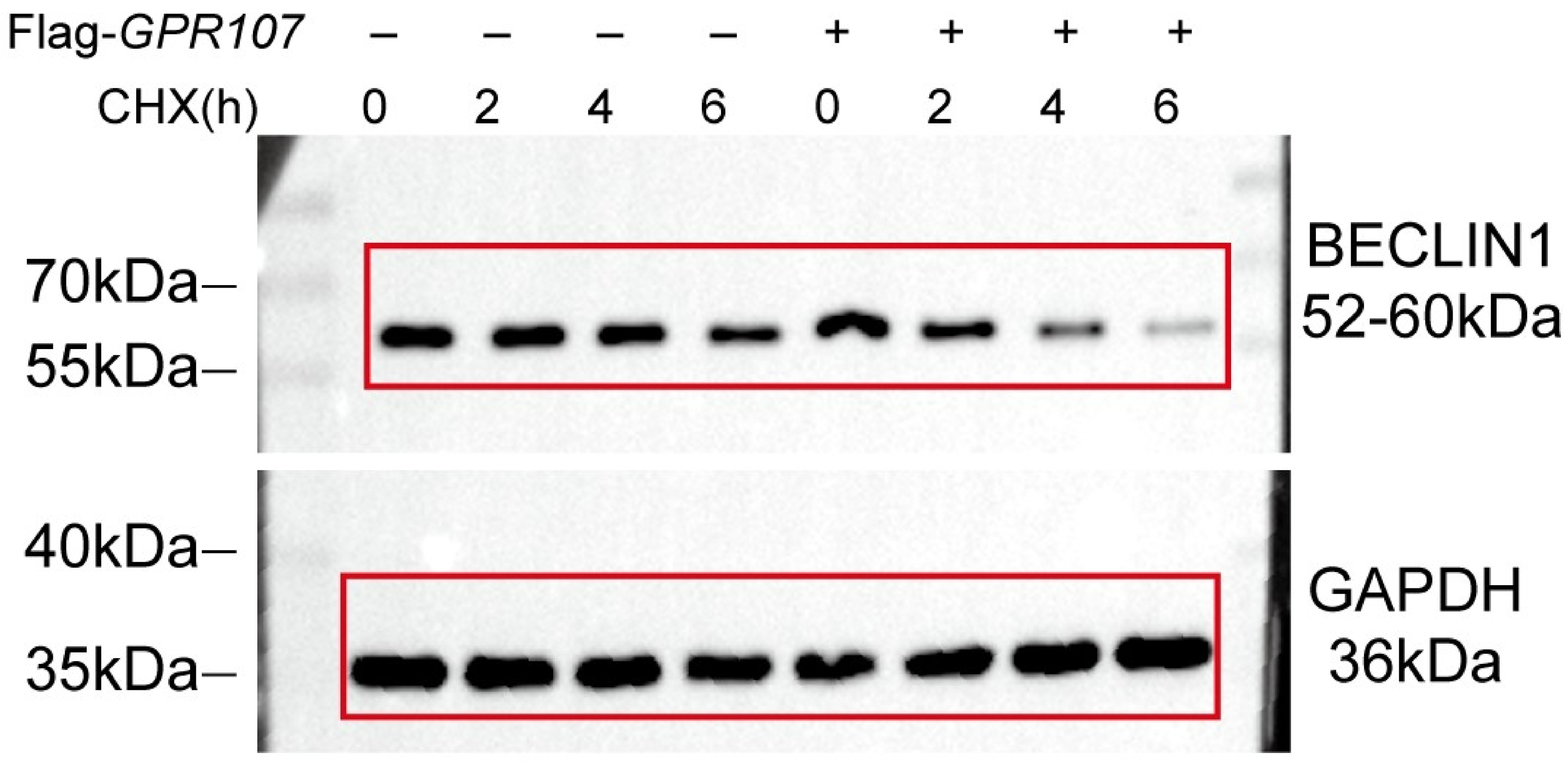

Fig4E

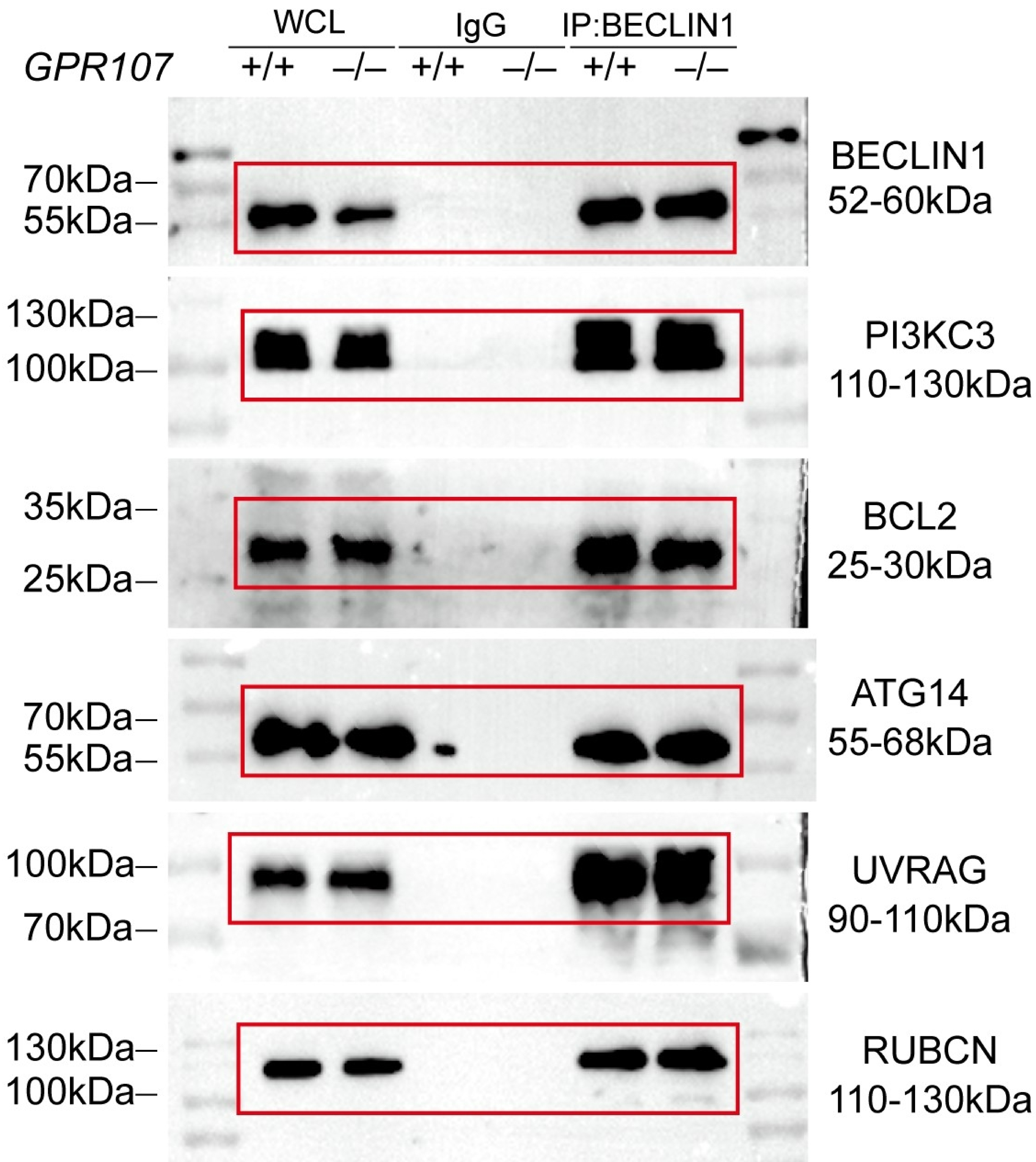

Fig4H

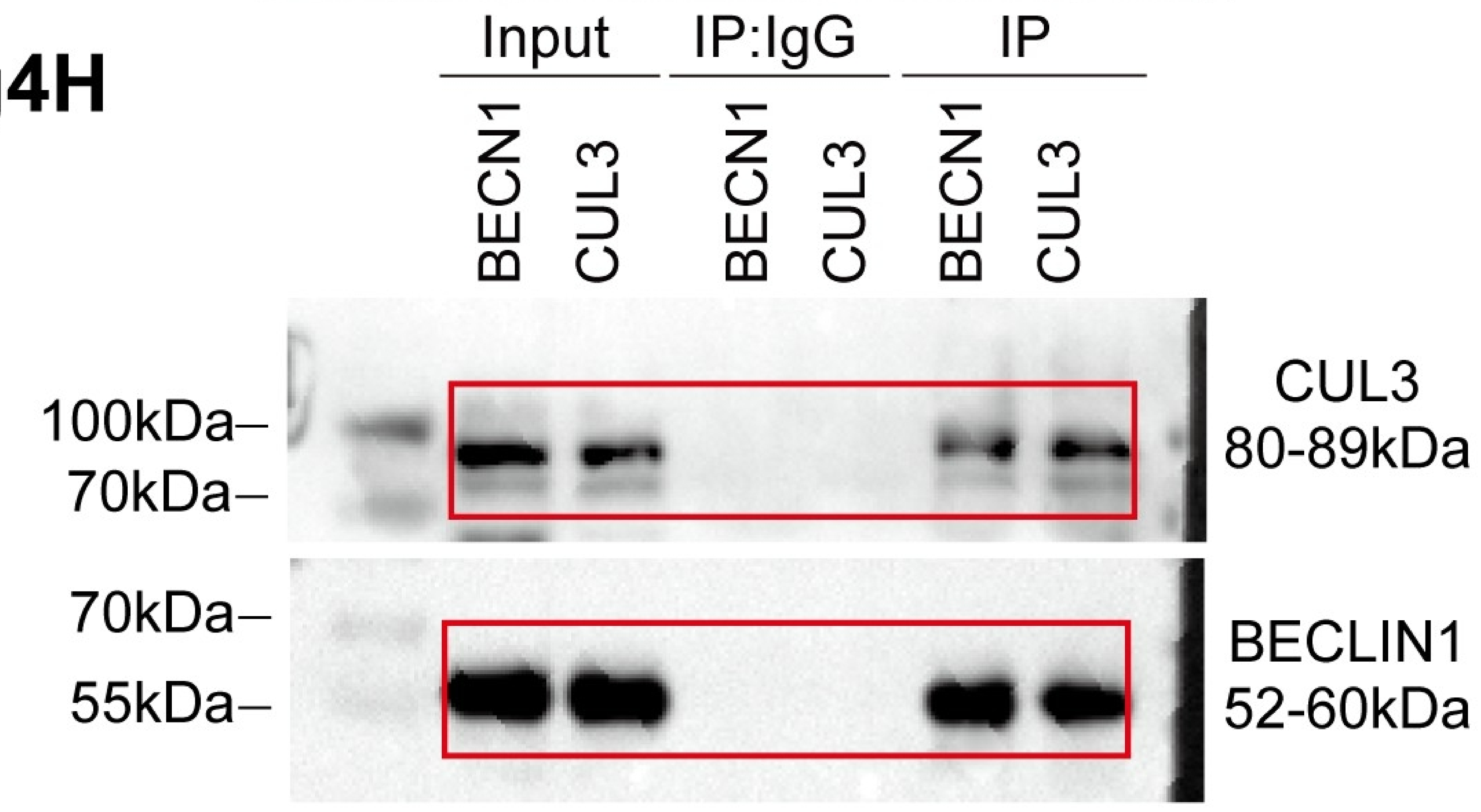

Fig4I

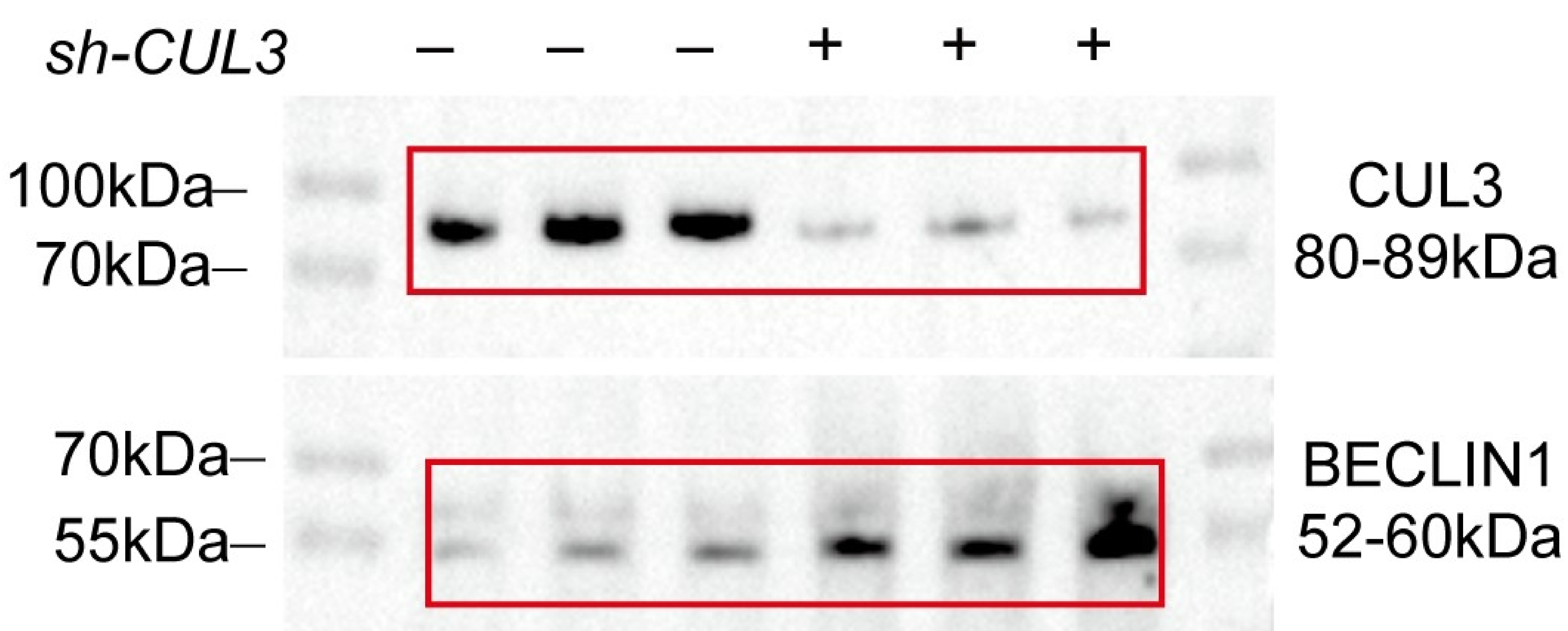

Fig5A

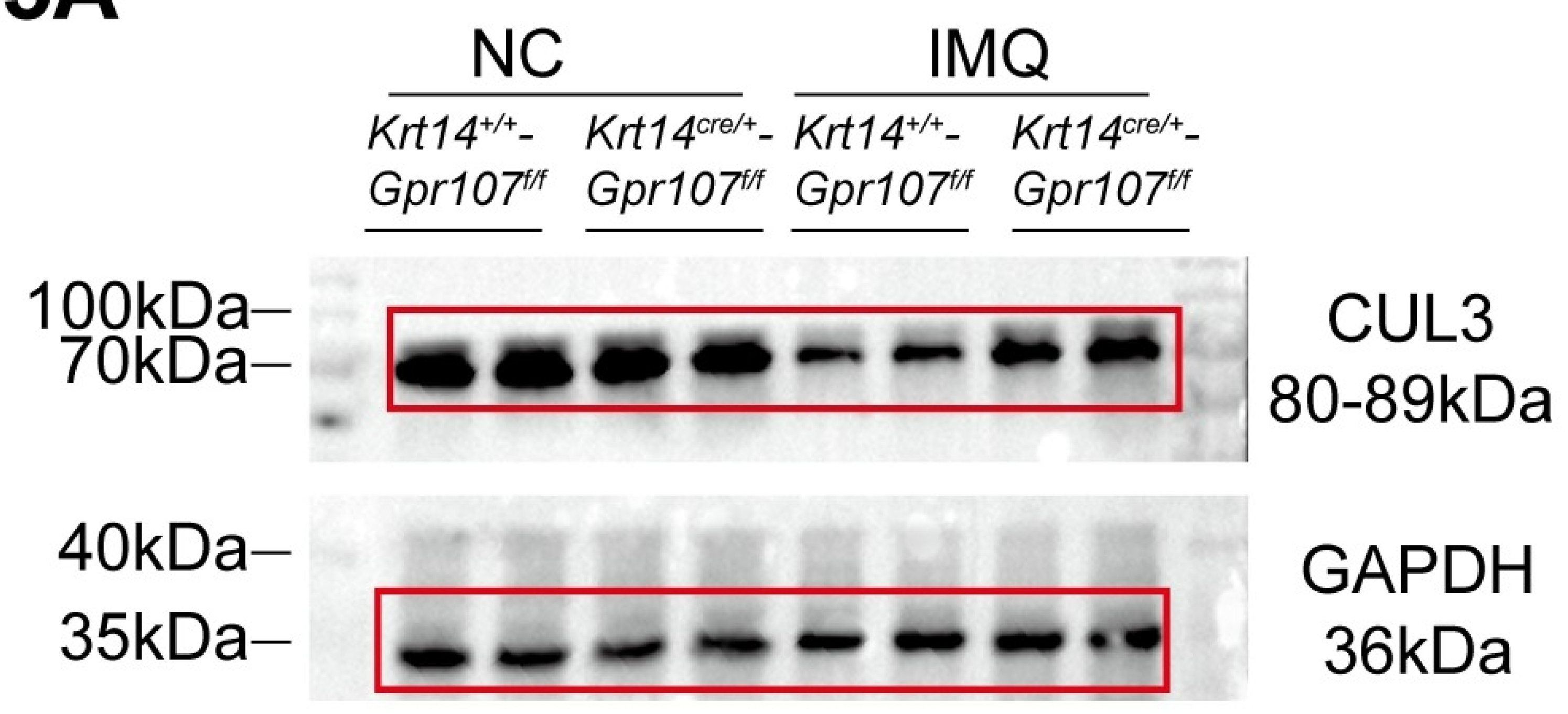

Fig5B

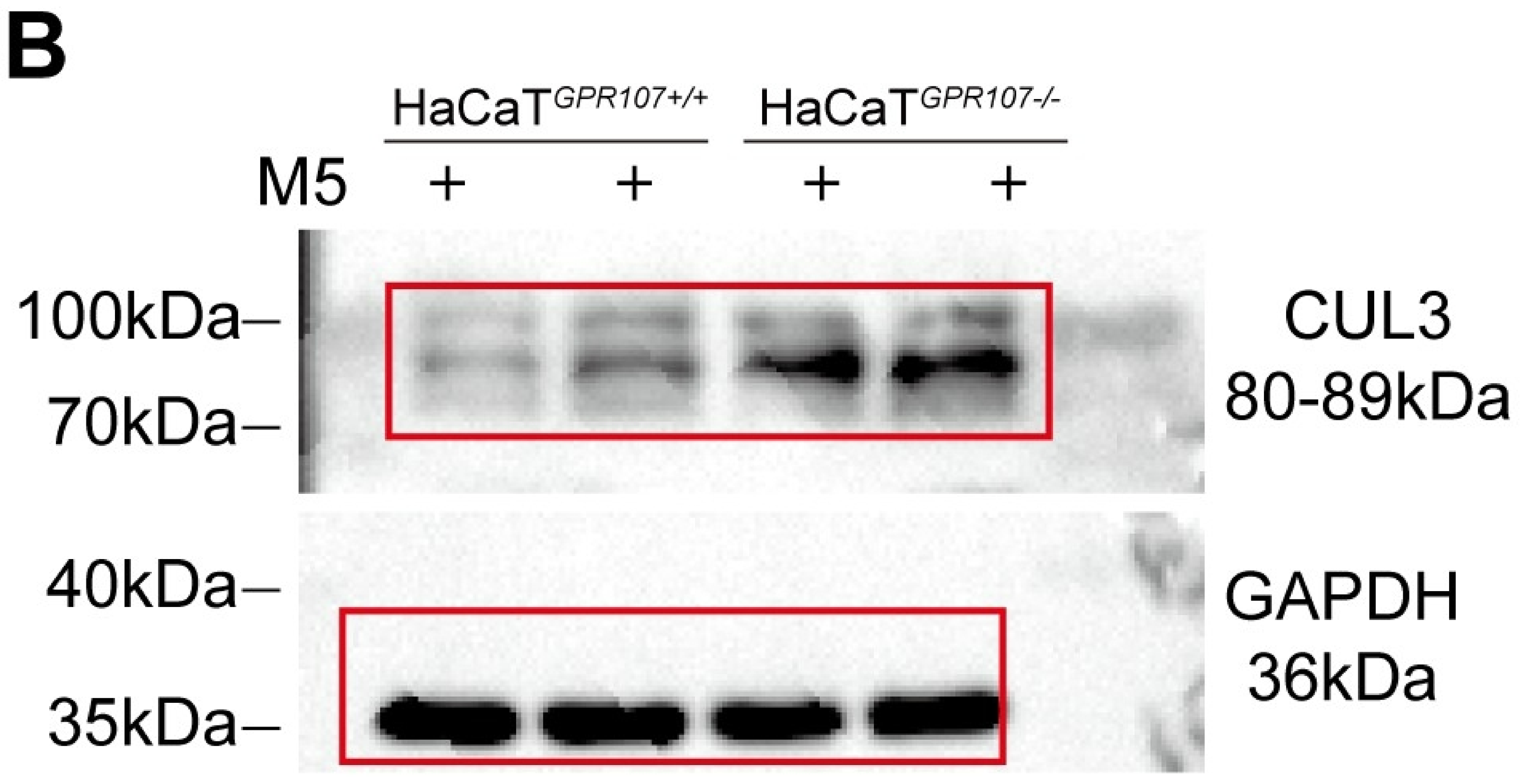

Fig5C

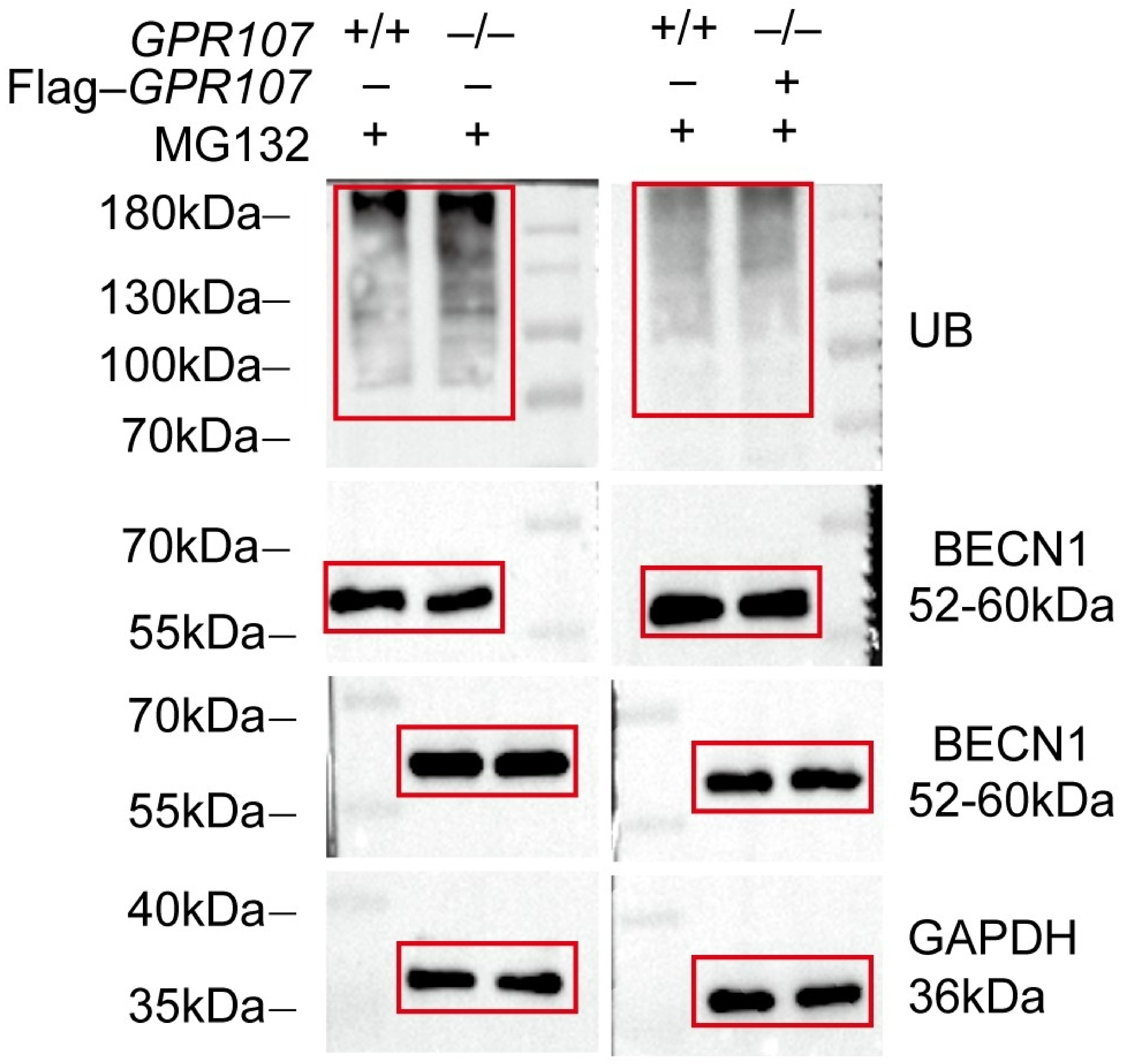

Fig5D

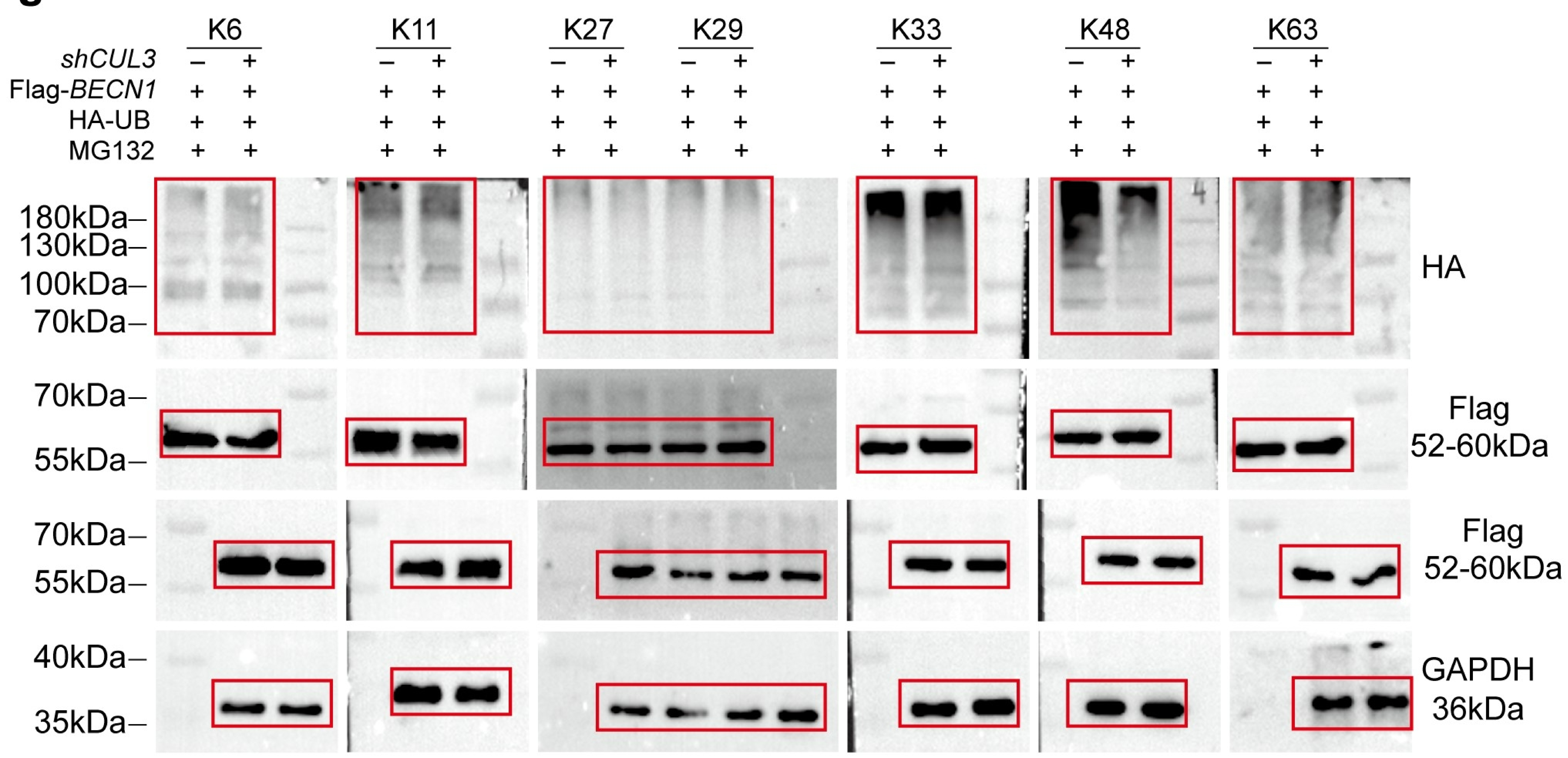

Fig5F

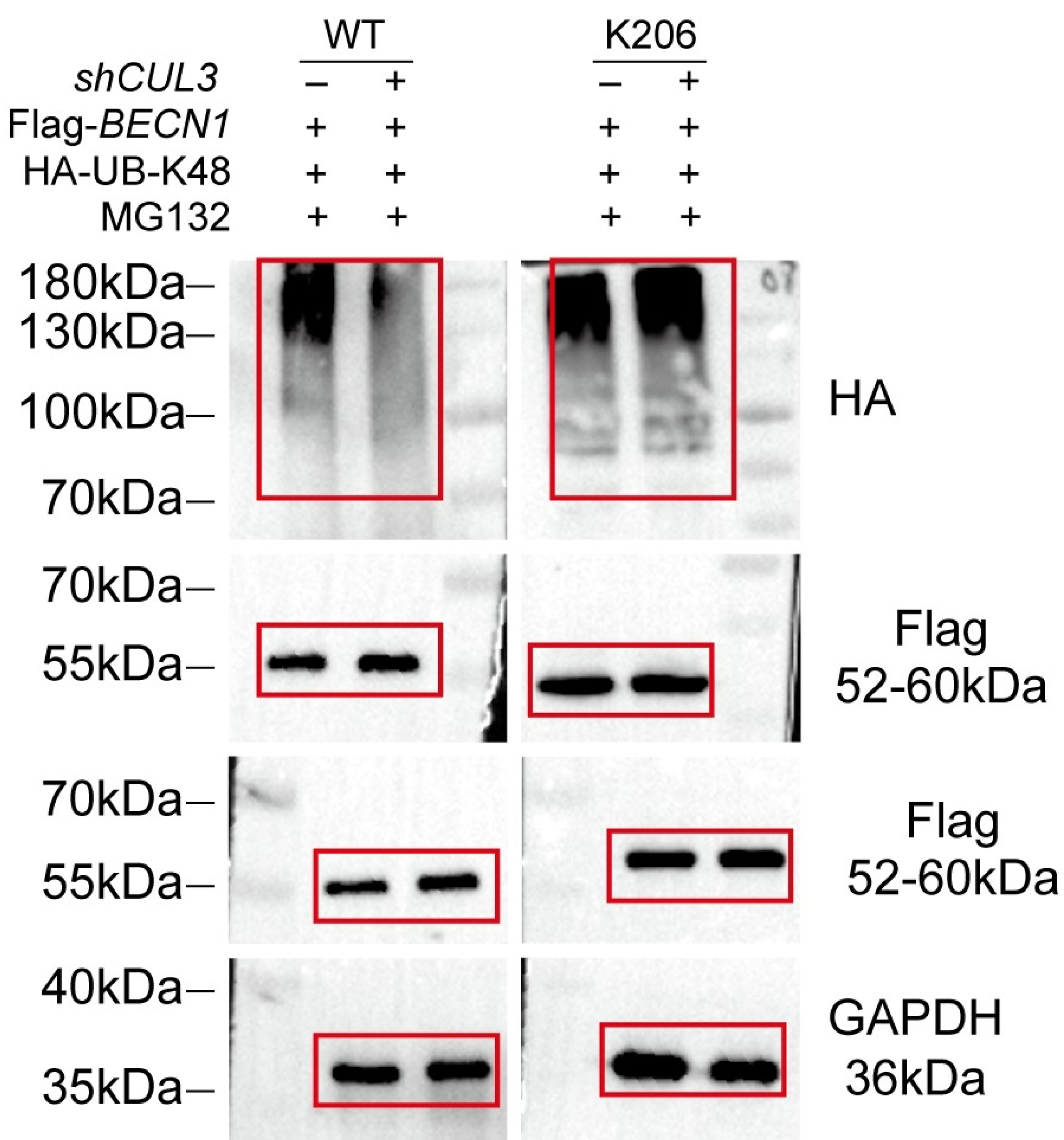

Fig6C

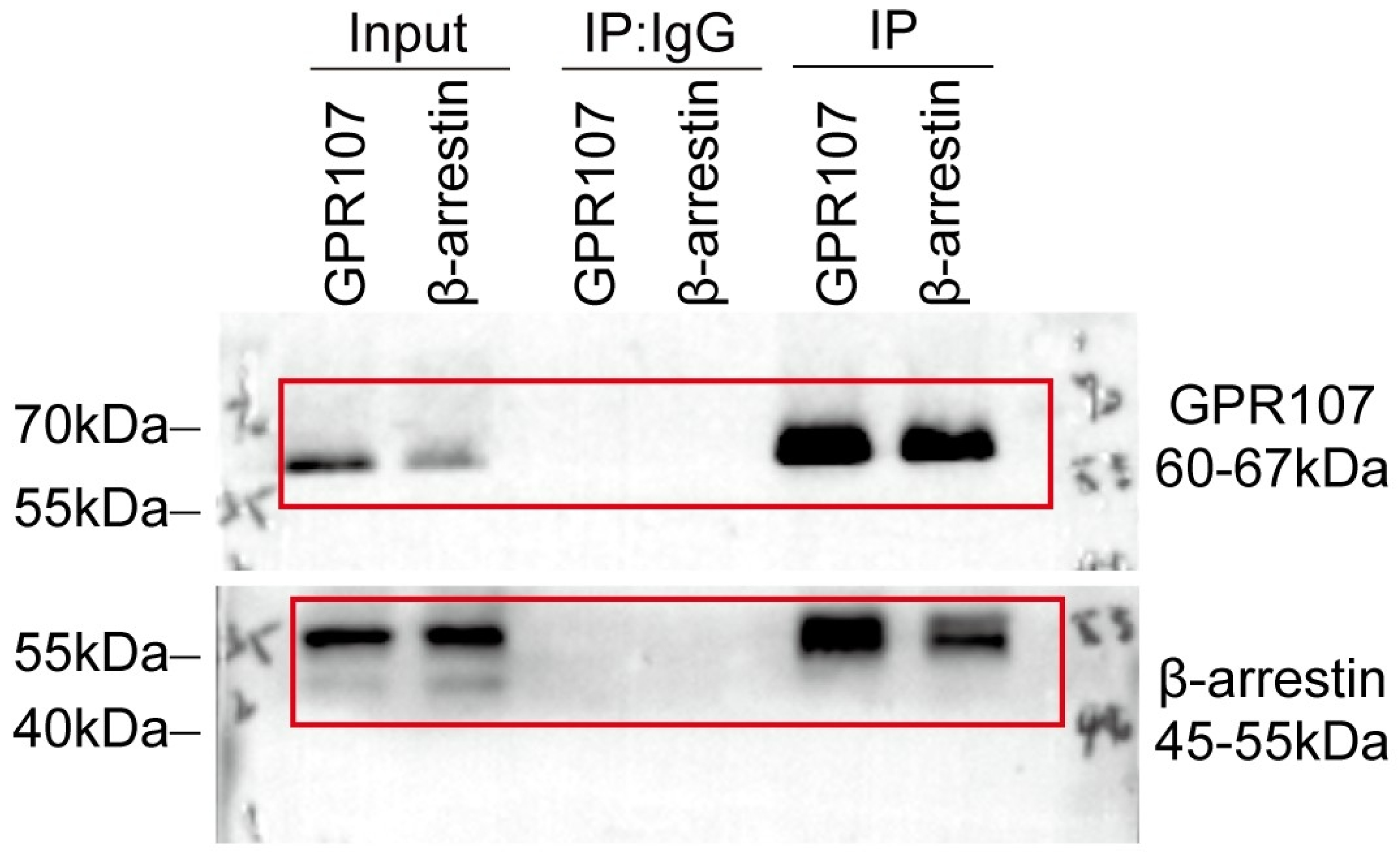

Fig6E

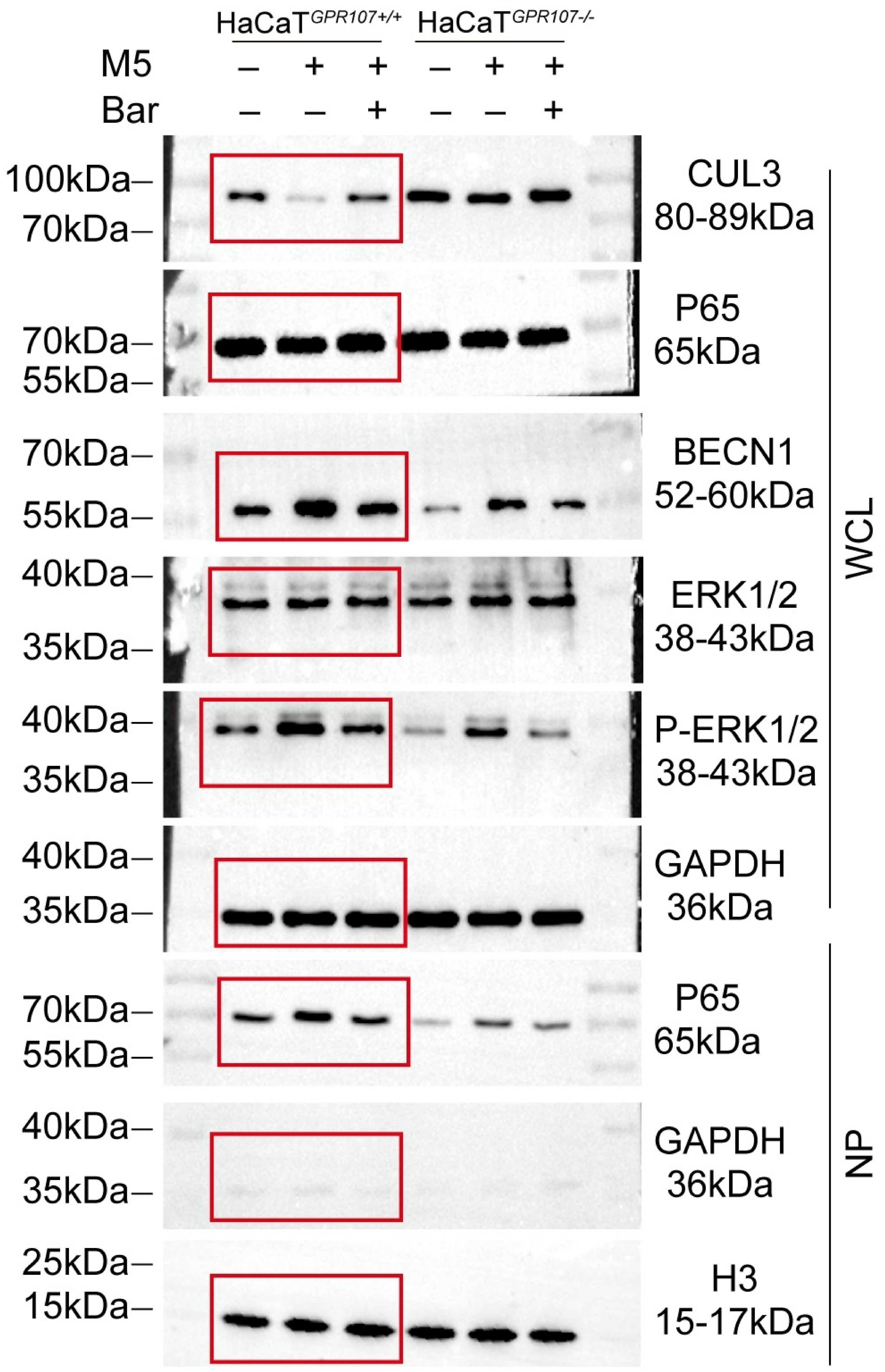

Fig7I

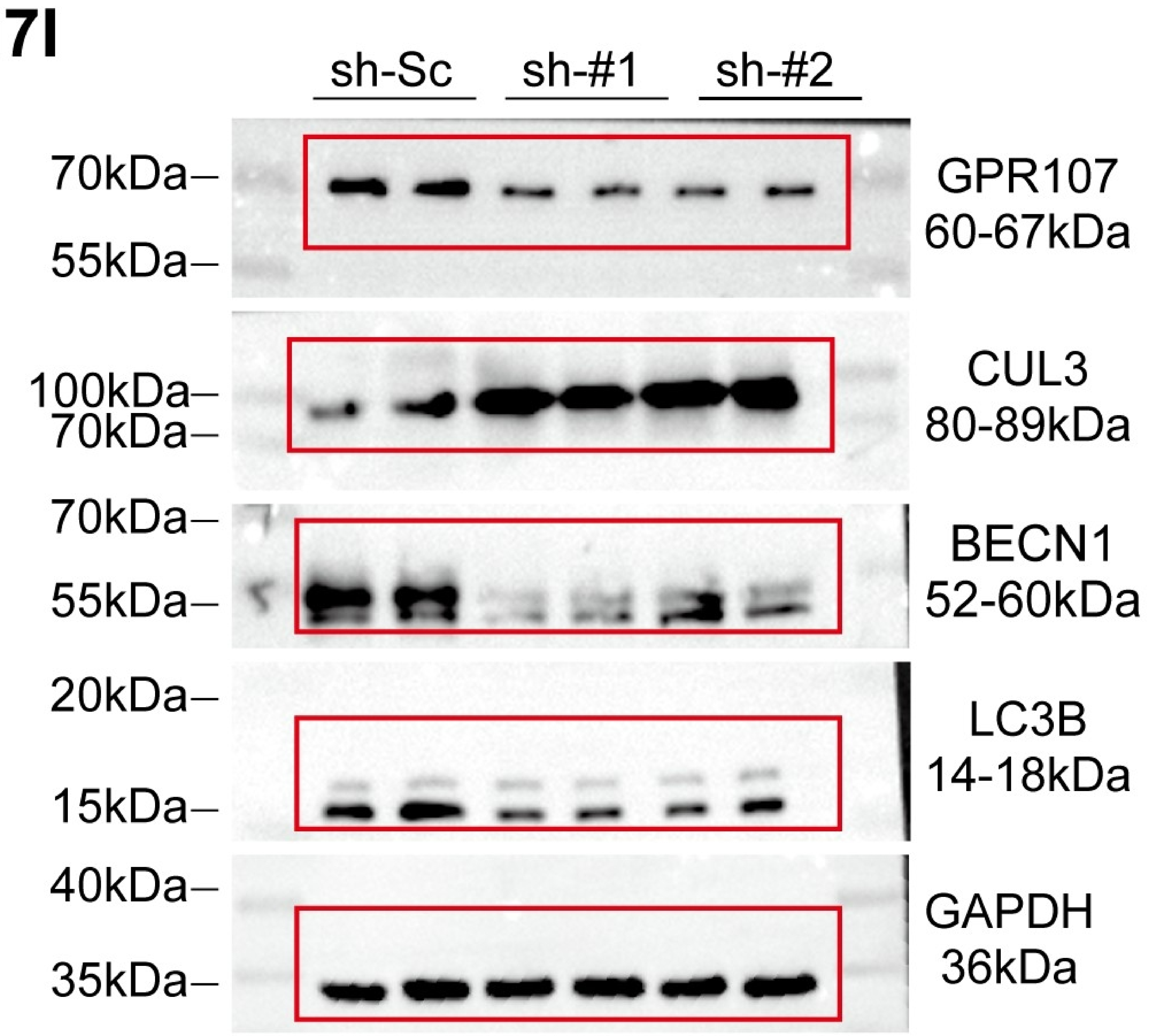

Supplementary Figure2E

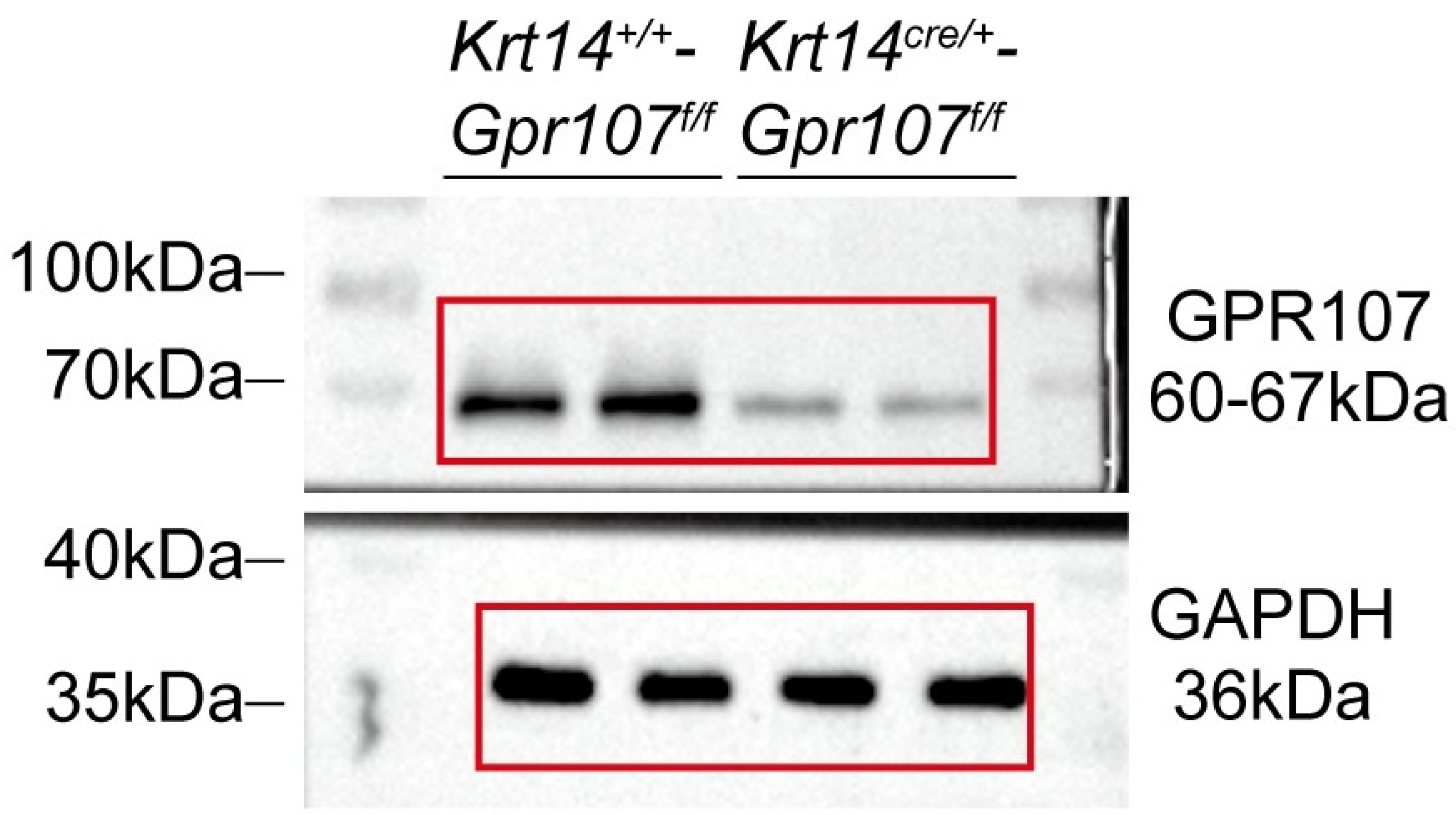

Supplementary Figure2F

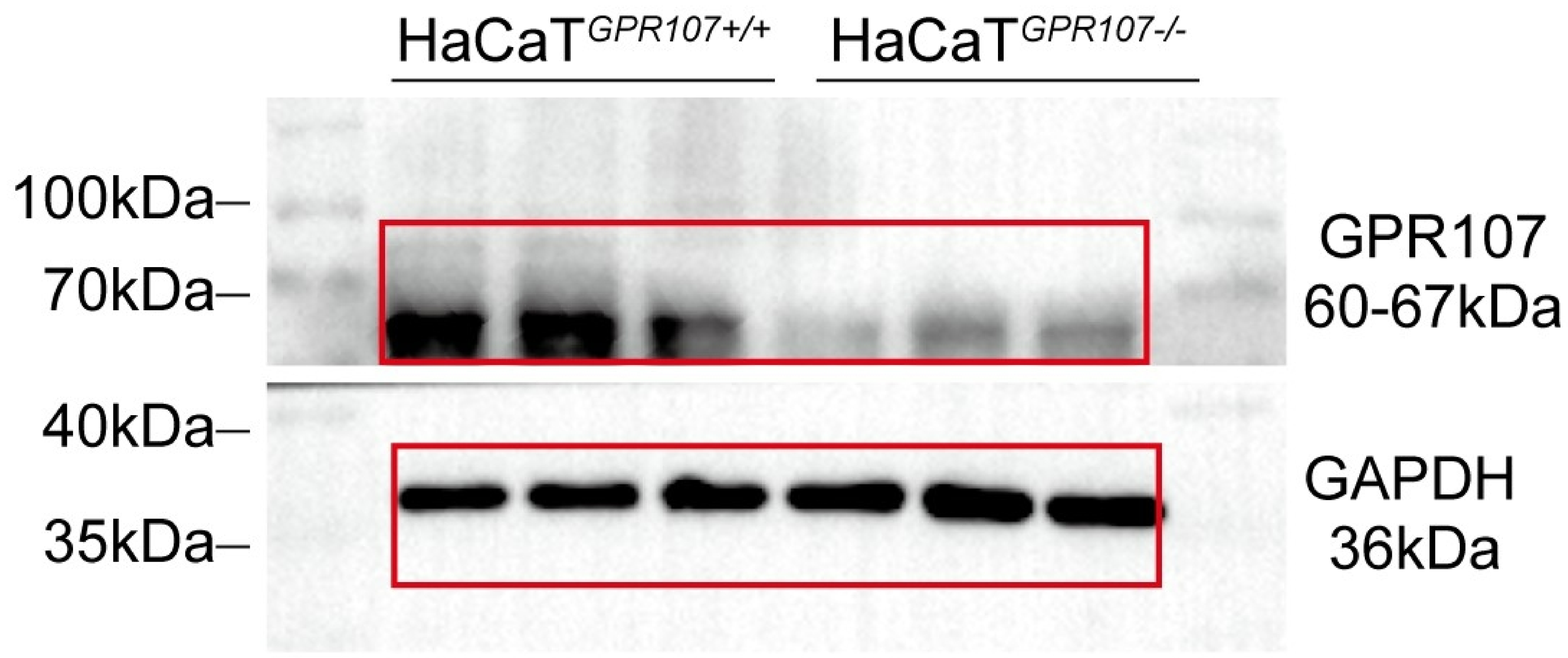

Supplementary Figure5A

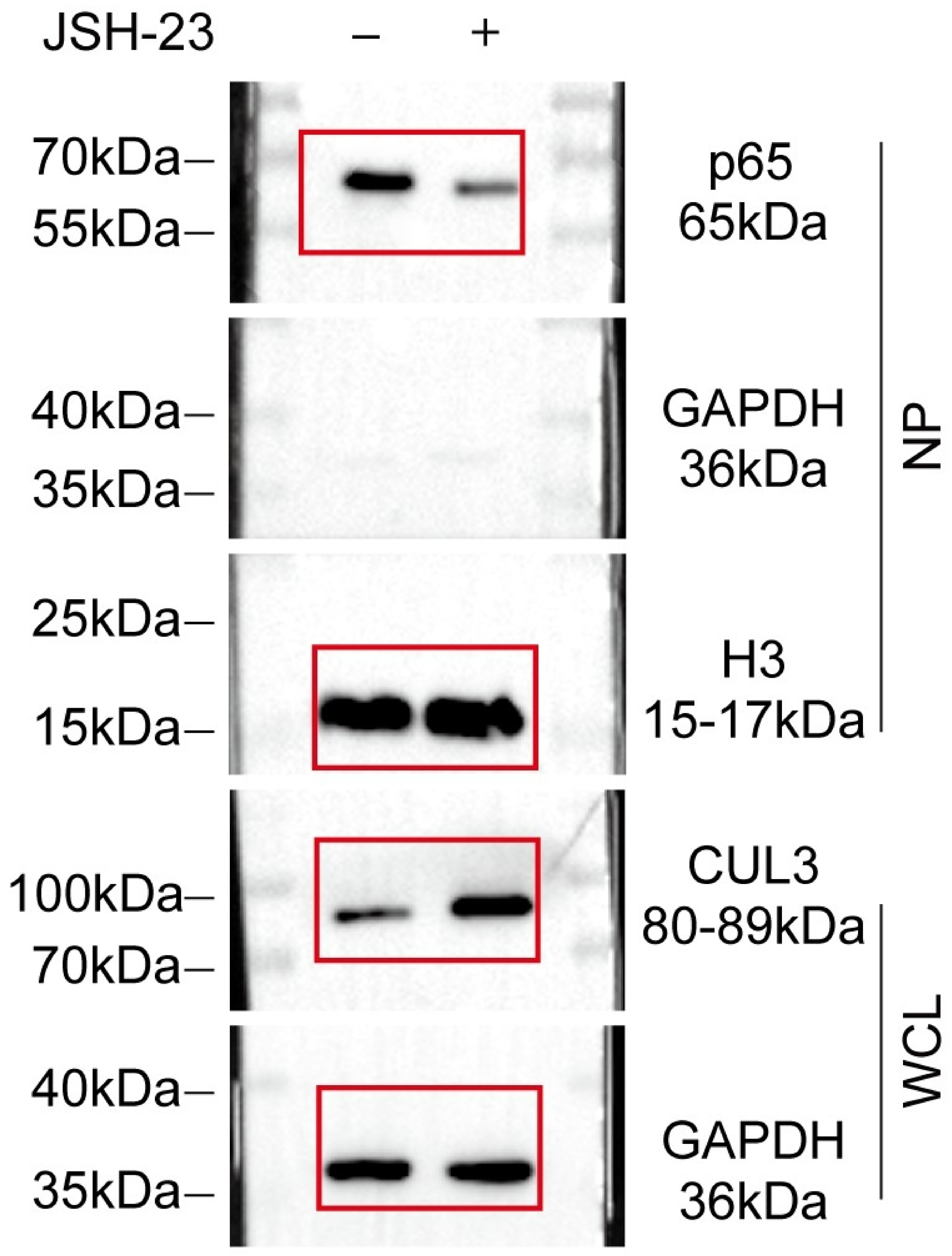

Supplementary Figure5C

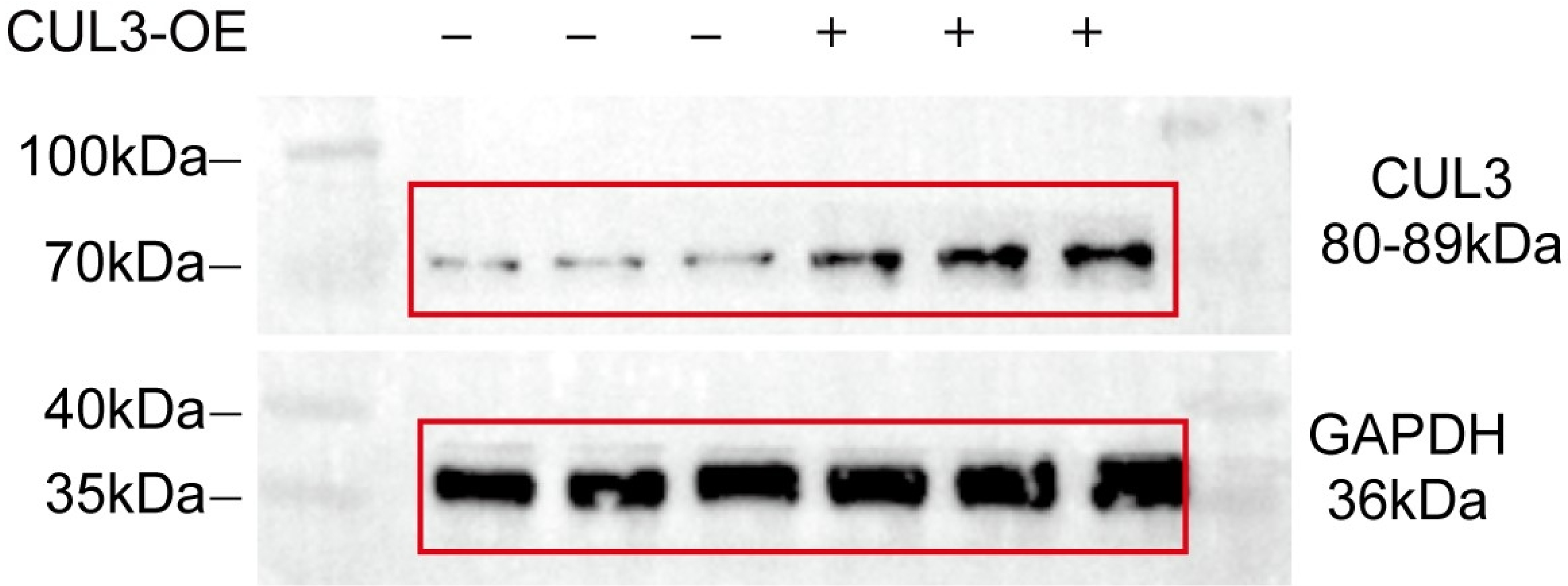

Supplement: Supplementary file 1 [file 41419_2026_9038_MOESM9_ESM.pdf]
