# Supplementary material for: GPR107 promotes autophagy secretion in psoriatic keratinocytes by inhibiting BECN1 K48-linked ubiquitination
Source: Cell Death Dis. 2026 Jun 26;17(1):796. doi: 10.1038/s41419-026-09038-9 (PMC13572499; doi:10.1038/s41419-026-09038-9)
Supplement: Supplementary file 2 [file 41419_2026_9038_MOESM1_ESM.docx]

**Supplementary Information**

GPR107 Promotes Autophagy Secretion in Psoriatic Keratinocytes by Inhibiting BECN1 K48-linked Ubiquitination

Kainan Liao *et al*.

**Supplementary Materials and methods**

**Patients and skin specimens**

All patients and healthy donors included in this study (5 psoriasis patients and 5 healthy donors) were recruited from the Department of Dermatology, First Affiliated Hospital of Anhui Medical University. The enrolled psoriasis patients were first-visit cases who had not received topical treatment for at least two weeks or systemic therapy for at least one month prior to skin biopsy. Skin biopsies (6 mm) were obtained from lesional sites of psoriasis patients, while biopsies from non-inflamed skin of healthy donors served as controls. All specimens were fixed in 4% paraformaldehyde for tissue sectioning.

**Mice, imiquimod (IMQ)-induced psoriasis model, and treatment.**

C57BL/6J mice were obtained from Nanjing Gem Pharma Co. (Nanjing, China). *Gpr107^flox/flox^* mice and *Krt14cre* mice, both on a C57BL/6 background, were purchased from Cyagen (Wuhan, China). The generation of keratinocyte-specific *Gpr107* knockout mice is shown in Fig S1. The genotyping primers for PCR identification are listed in Table S1. All mice were bred and maintained under specific pathogen-free conditions at the Animal Center of the School of Basic Medical Sciences, Anhui Medical University (AHMU). No statistical methods were used to predetermine sample sizes. Animal sample sizes were chosen based on our previous experience with this model to ensure adequate reproducibility. No animals or samples were excluded from the analysis. Investigators were blinded to group allocation during outcome assessment.

Mice matched for age and weight were randomly allocated to different experimental groups prior to IMQ application. IMQ-induced psoriasis model was established as previously described[1]. Briefly, six-week-old male mice received a daily dose of 60 mg of 5% IMQ cream (Sichuan Meixinda, China. Cat# H20030128) applied to a 4 cm² shaved area on the dorsal skin, or 20 mg of 5% IMQ cream applied to one side ear skin for consecutive 6 days. All mice were euthanized on day 6, after which tissue samples were collected and the psoriasis area and severity index (PASI) scores were assessed. The PASI scores, which consisted of measurements or inflammation, including skin erythema, scaling and thickness, was usually used to monitor and grade the severity of the psoriasis-like lesions on day 7. Each parameter was scored independently on a five-point scale from 0 to 4, namely: 0, none; 1, slight; 2, moderate; 3, marked; and 4, very marked[2].

To silence *Gpr107* expression in IMQ-treated skin, a viral supernatant containing 5.0-6.0×10^7^ transducing units (shRNA-Scramble, shRNA-*Gpr107*#1, and shRNA-*Gpr107*#2) was applied to the dorsal lesions, and 1.0-2.0×10^7^ transducing units were applied to the ear lesions, 6 hours after the daily application of 60 mg of 5% IMQ cream. The targeted shRNA sequences were listed in Table S1.

**Cell culture and treatments**

The immortalized human keratinocyte cell line (HaCaT) was obtained from the National Biomedical Cell Resource Bank (Beijing, China). Cells were cultured in Dulbecco's Modified Eagle Medium (DMEM) (Gibco) supplemented with 10% fetal bovine serum (Gibco) and 1% penicillin–streptomycin (Thermo Fisher Scientific) at 37℃ in a humidified atmosphere containing 5% CO_2_. Normal human epidermal keratinocytes (NHEKs) were purchased from Wanwu Biotechnology (Hefei, China) and cultured in Keratinocyte Growth Medium (KGM) supplemented with growth additives and 1% penicillin–streptomycin under the same conditions. All cell lines were authenticated by STR profiling and tested for mycoplasma contamination.

For M5 stimulation experiments aimed at inducing a psoriatic-like inflammatory state in keratinocytes, cells were treated with a cytokine mixture consisting of recombinant human IL-1α (MedChemExpress, Cat#HY-P72380), oncostatin M (OSM, Cat#HY-P70465), IL-17A (Cat#HY-P76418), IL-22 (Cat#HY-P7039), and TNF-α (Cat#HY-P70426A), each at a final concentration of 10 ng/mL, for 24 hours. Other reagents were used to inhibit specific cellular processes, as described in the corresponding experiments of this study, including a proteasome inhibitor (MG132, Cat#HY-13259), an autophagy inhibitor (Bafilomycin A1, Baf A1, Cat#HY-100558), a protein synthesis inhibitor (Cycloheximide, CHX, Cat#HY-12320), an inhibitor of p65 nuclear translocation (JSH-23, Cat#HY-13982), and a β-arrestin inhibitor (Barbadin, Bar, Cat#HY-119706).

Transfection was carried out using Lipofectamine 3000 reagent (Thermo Fisher Scientific, Cat#L3000075) to overexpress or silence target genes in keratinocytes, following the manufacturer’s instructions. For overexpression, plasmids included Flag-*GPR107*, Flag-*BECN1*, HA-Ub, and HA-K48-Ub, while gene knockdown was achieved using sh*BECN1* or sh*CUL3*. The targeted shRNA sequences were listed in Table S1. Cells were harvested 48 h post-transfection for subsequent experiments.

**Western blot analysis**

Western blot analysis was performed as described elsewhere[1, 3]. In brief, cell or skin lysate proteins were separated by SDS-PAGE, transferred onto PVDF membranes, and analyzed by immunoblotting. Band intensities were quantified using ImageJ, and statistical analyses and graph generation were performed with GraphPad Prism 8.4.0.

The antibodies used for immunoblotting analysis included: anti-LC3B (Cat#2775), anti-PIK3C3/Vps34 (Cat#4263), anti-Bcl-2 (Cat#15071), anti-Flag (Cat#14793), anti-p65 (Cat#3033), anti-Erk1/2 (Cat#4695), anti-phosphorylated Erk1/2 (Cat#4370) from Cell Signaling Technology; anti-GAPDH (Cat#60004-1-Ig), anti-GPR107 (Cat#25076-1-AP), anti-BECN1 (Cat#11306-1-AP), anti-ATG14 (Cat#19491-1-AP), anti-UVRAG (UV Radiation Resistance-Associated Gene, Cat#84901-1-RR), anti-ubiquitin (Cat#10201-2-AP), anti-HA tag (Cat#51064-2-AP), anti-CUL3(Cullin, Cat#11107-1-AP), anti-H3 (Cat#17168-1-AP) from Proteintech; anti-RUBCN/rubicon (RUN domain and Cysteine-rich domain containing Becn1-interacting protein, Cat#ab92388) and anti-β-arrestin (Cat#ab32099) from Abcam.

**RNA-seq analysis and quantitative RT-PCR analysis (RT-qPCR)**

Total RNA was extracted from cells or tissues using TRIzol reagent (Thermo Fisher Scientific, Cat#15596018), and cDNA was synthesized with a reverse transcription kit according to manufacturer's requirements (Vazyme, Nanjing, China). The construction and sequencing of the library were performed by Lianchuan Biotechnology Corporation (Hangzhou, China). The raw RNA sequencing data have been deposited in the NCBI BioProject database under the accession number PRJNA1468314. RT-qPCR was then performed on a LightCycler 480 II system (Roche Life Science, Switzerland) using SYBR Green Master Mix (Vazyme, Nanjing, China) and gene-specific primers. The primer sequences are listed in Table S1.

**Hematoxylin and eosin (H&E) staining**

Dorsal skin tissues from mice were fixed in 4% paraformaldehyde for at least 24 hours, embedded in paraffin, and sectioned at a thickness of 4 µm. Sections were stained with a H&E staining kit (Solarbio, Shanghai, China). Epidermal thickness was quantified using the TissueFAXS Plus ST system (TissueGnostics, Austria).

**Immunohistochemistry**

Immunohistochemistry was performed as previously described[1, 3]. Briefly, tissue sections were deparaffinized, rehydrated, and incubated with 3% hydrogen peroxide to quench endogenous peroxidase activity. Antigen retrieval was carried out by microwaving the sections in 1% citrate buffer for 15 min, followed by blocking with 1.5% normal serum. Sections were then incubated with primary antibodies, and immunostaining was visualized using biotinylated anti-IgG, DAB chromogenic detection, and hematoxylin counterstaining. Primary antibodies used for immunohistochemistry analysis included: anti-GPR107 (Proteintech, Cat#25076-1-AP), anti- Loricrin (Proteintech, Cat# 55439-1-AP), anti-LC3B (Cell Signaling Technology, Cat#2775), anti-Ki-67 (Proteintech, Cat#27309-1-AP), and anti-Cytokeratin 1 (K1, Proteintech, Cat#82972-1-RR). Images were acquired using a TissueFAXS Plus ST system (TissueGnostics, Austria) and analyzed with Image-Pro Plus 6.0 software.

**Immunofluorescence Staining**

Immunofluorescence staining was performed as previously described[1, 3]. In brief, keratinocytes were fixed with 4% paraformaldehyde, permeabilized with 0.2% Triton X-100, washed with PBS, and blocked with 5% bovine serum albumin. Primary antibodies, including anti-GPR107 (Proteintech, Cat#25076-1-AP) and anti-β-arrestin1 (Abcam, Cat# ab32099), as well as corresponding secondary antibodies, were diluted in blocking buffer, and fluorescence images were acquired using an LSM 980 microscope equipped with Airyscan 2.

**Co-immunoprecipitation (Co-IP)**
Co-IP was performed as previously described[1]. In brief, cells were lysed in immunoprecipitation cell lysis buffer, and the lysates were centrifuged to obtain the supernatant, which was incubated overnight at 4 ℃ with the primary antibody conjugated to protein G agarose, according to the manufacturer’s instructions (Thermo Fisher Scientific, Cat# 26147). After five washes with Tris-buffered saline, the immunoprecipitates and input samples were separated by SDS–PAGE and analyzed by immunoblotting. Primary antibodies for Co-IP included anti-BECN1 (Proteintech, Cat#11306-1-AP), anti-CUL3 (Proteintech, Cat#11107-1-AP), anti-Flag (Cell Signaling Technology, Cat#14793), anti-GPR107 (Proteintech, Cat#25076-1-AP), and anti-β-arrestin (Abcam, Cat#ab32099).

**Transmission electron microscopy**

Sample processing was performed at the Electron Microscopy Center of Anhui Medical University, as previously described[4]. Images were captured and analyzed, and autophagic vacuoles were quantified using a transmission electron microscope (Thermo Scientific).

**Microarray data processing and validation of *GPR107* expression**

The gene expression datasets GSE14905 and GSE117468 were obtained from the GEO database ([https://www.ncbi.nlm.nih.gov/geo/](https://www.ncbi.nlm.nih.gov/geo/)). Specifically, GSE14905 includes 33 psoriatic lesional and 28 non-lesional skin samples, while GSE117468 comprises 216 samples from the brodalumab-treated group and 62 from the placebo group. After acquiring the raw data, we validated the mRNA expression of *GPR107* in these cohorts using R software.

**Statistical analysis**

Data are presented as mean ± standard deviation (SD). Normality was assessed using appropriate tests. For normally distributed data, statistical significance was determined by unpaired two-tailed Student’s t-test or one-way ANOVA using GraphPad Prism 8 software. For data not conforming to a normal distribution, non-parametric tests including the Wilcoxon test or Kruskal–Wallis test were applied. A *p* value  <  0.05 was considered statistically significant.

**Reference**

1.	Zhao W, Liao K, Song W, Wang J, Cai C, Zhou F, *et al*. Chaperone-mediated autophagy dysfunction in imiquimod-induced psoriasiform dermatitis. Autophagy Rep. 2025;4(1):2544061.

2.	Chen H, Lu C, Liu H, Wang M, Zhao H, Yan Y, *et al*. Quercetin ameliorates imiquimod-induced psoriasis-like skin inflammation in mice via the NF-κB pathway. Int Immunopharmacol. 2017;48:110-7.

3.	Tu Z, Zhang S, Zhou G, Zhou L, Xiang Q, Chen Q, *et al*. LMO4 Is a Disease-Provocative Transcription Coregulator Activated by IL-23 in Psoriatic Keratinocytes. J Invest Dermatol. 2018;138(5):1078-87.

4.	Xu D, Tong Z, Yang P, Chen Q, Wang S, Zhao W, *et al*. G protein-coupled receptor 107 deficiency promotes development of diabetic nephropathy. Mol Biomed. 2025;6(1):10.

**Supplementary Table S1: List of Oligonucleotides.**

| **Oligos** | **Sequence（5’ to 3’）** | **Aims** |
| --- | --- | --- |
| *Gpr107flox* | FW: CAGTGTTTACCTTTTCTGCACTTCA | PCR identification of keratinocyte-specific *Gpr107* knockout mice |
|  | RW: TCCAGACTTTGCTTTAACACTGCT |  |
| *Krt14-Cre* | FW: CATATTGGCAGAACGAAAACGC |  |
|  | RW: CCTGTTTCACTATCCAGGTTACGG |  |
| shRNA-Scramble | TTCTCCGAACGTGTCACGT | Knockdown the target genes |
| shRNA-*Gpr107*#1 | AGAAGAAAAGCCAUCGUUCUU |  |
| shRNA-*Gpr107*#2 | UAGUAAUGUCUGAAAAGCCUC |  |
| sh*BECN1* | UGGAAUGGAAUGAGAUUAATT |  |
| sh*CUL3* | GCTTGGAATGATCATCAAACA |  |
| Human *GAPDH* | FW: TCACTGCCACTCAGAAGACTGT | qPCR detection |
|  | RW: CGTTCAGCTCTAGGATGACCTT |  |
| Human *CCL20* | FW: TGCTGTACCAAGAGTTTGCTC |  |
|  | RW: CGCACACAGACAACTTTTTCTTT |  |
| Human *CXCL1* | FW: CGCCCAAACCGAAGTCATAG |  |
|  | RW: CGCCCAAACCGAAGTCATAG |  |
| Human *CXCL2* | FW: TTCACAGTGTGTGGTCAACAT |  |
|  | RW: TCTCTGCTCTAACACAGAGGGA |  |
| Human *S100A7* | FW: ACGTGATGACAAGATTGACAAGC |  |
|  | RW: GCGAGGTAATTTGTGCCCTTT |  |
| Human *BECN1* | FW: CCATGCAGGTGAGCTTCGT |  |
|  | RW: GAATCTGCGAGAGACACCATC |  |
| Mouse *Gapdh* | FW: ACCCTTAAGAGGGATGCTGC | qPCR detection |
|  | RW: CCCAATACGGCCAAATCCGT |  |
| Mouse *Ccl20* | FW: GCCTCTCGTACATACAGACGC |  |
|  | RW: CCAGTTCTGCTTTGGATCAGC |  |
| Mouse *Cxcl1* | FW: ACTGCACCCAAACCGAAGTC |  |
|  | RW: TGGGGACACCTTTTAGCATCTT |  |
| Mouse *Cxcl2* | FW: CCAACCACCAGGCTACAGG |  |
|  | RW: GCGTCACACTCAAGCTCTG |  |
| Mouse *S100a7* | FW: TGCTCTTGGATAGTGTGCCTC |  |
|  | RW: GCTCTGTGATGTAGTATGGCTG |  |
| Mouse *Il17* | FW: CTCAGACTACCTCAACCGTTCC |  |
|  | RW: ATGTGGTGGTCCAGCTTTCC |  |
| Mouse *Defb4* | FW: TTTAACTCCCTTGGCGCAAAA |  |
|  | RW: CTTTCCCTCCGCATTGACAC |  |

***SUPPLEMENTARY FIGURE LEGENDS***

**Figure S1. Upregulation of GPR107 in human psoriatic lesions and IMQ-induced psoriasiform skin.**

**A** Immunohistochemical analysis of GPR107 in healthy control and psoriatic skin (from Fig. 1C). Scale bars: upper panels = 100 μm; lower panels = 200 μm. **B** Immunohistochemistry analysis of loricrin expression in mouse control skin (vaseline-treated, NC) and IMQ-induced psoriasiform lesions (IMQ). Scale bars = 100 μm. **C** qRT-PCR analysis of *Il17*, *S100a7*, and β4-defensin (*Defb4)* mRNA levels in the skin of mouse control skin (vaseline-treated, NC) and IMQ-induced psoriasiform lesions (IMQ). Data are presented as mean ± SD (n = 5). *** *p* < 0.001. **D** Immunohistochemical staining of GPR107 in control and IMQ-induced psoriasiform skin (from Fig. 1E). Scale bars: upper panels = 100 μm; lower panels = 200 μm.

**Figure S2. Targeted disruption of GPR107 gene loci in keratinocytes.**

**A** Schematic diagram of the breeding strategy employed to generate keratinocyte-specific *Gpr107* knockout mice. **B** Schematic diagram of the targeting strategy utilized to generate *Gpr107* knockout in gene clusters. **C** and **D** Genotyping PCR validating *Gpr107^flox/flox^* and *Krt14-Cre* mouse genotypes. **E** Western blot analysis of GPR107 expression in keratinocytes from control mice and keratinocyte-specific *Gpr107* knockout mice. **F** Western blot analysis of GPR107 expression in HaCaT^G^*^PR107^*^+/+^ and HaCaT*^GPR107^*^-/-^ cells.

**Figure S3. Immunohistochemical staining of Ki-67 and keratin 1 in control and IMQ-induced psoriasiform skin**

**A** Immunohistochemistry analysis of Ki-67-positive cells in the skin of *Krt14^+/+^-Gpr107^f/f^* and *Krt14^Cre/+^-Gpr107^f/f^* mice treated with vaseline (NC) or IMQ for 6 days (from Fig. 2E).**B** Immunohistochemistry staining of keratin 1 (K1) in dorsal skin of *Krt14^+/+^-Gpr107^f/f^* and *Krt14^Cre/+^-Gpr107^f/f^* mice treated with vaseline（NC）or IMQ for 6 days (from Fig. 2G). Scale bars: upper panels = 100 μm; lower panels = 200 μm.

**Figure S4. M5 induces autophagy-dependent secretion in keratinocytes.**

**A**. Immunohistochemistry staining of LC3B in skin of healthy controls and psoriasis patients (from Fig 3A). Scale bars: left panels = 100 μm; right panels = 200 μm. **B**. Quantitative RT‑PCR analysis of cytokine and antimicrobial peptide transcripts in HaCaT cells. HaCaT keratinocytes were treated for 24 h with M5 (each at 10 ng/mL) alone, BafA1 (autophagy inhibitor,100 nM) alone, or the combination M5 + BafA1. Total RNA was isolated, reverse-transcribed, and subjected to quantitative real-time PCR. Relative mRNA levels of *CCL20*, *CXCL1*, *CXCL2*, and *S100A7* were measured and normalized to the housekeeping gene GAPDH. Data are presented as mean ± SD (n = 3). **p* < 0.05, ****p* < 0.001.

**Figure S5. Comparative Transcriptomics: GPR107 deficiency in HaCaT cells alters gene expression and KEGG pathways following M5 treatment.**

Total RNA was extracted from HaCaT^G^*^PR107^*^+/+^ and HaCaT*^GPR107^*^-/-^ cells treated with M5 for 24 hours respectively. The construction and sequencing of the library was performed by Lianchuan Biotechnology Corporation (Hangzhou, China). **A** Volcano plots from RNA-seq analysis show significantly upregulated chemokines, including *CCL20*, *CCL2*, *CXCL1*, *CXCL8*, *CXCL10*, *CXCL3*, and *CXCL11*, as well as antimicrobial peptides such as *S100A7*, *S100A8*, *S100A9*, *S100A1*2. **B** Analysis of differentially expressed genes in HaCaT^G^*^PR107^*^+/+^ and HaCaT*^GPR107^*^-/-^ cells treated with M5 for 24 hours. **C** Kyoto Encyclopedia of Genes and Genomes (KEGG) pathway analysis of the RNA-Seq data.

**Figure S6. Blocking p65 nuclear translocation upregulates CUL3 expression.**

**A** Western blot analysis of nuclear p65 and cytoplasmic CUL3 in HaCaT cells treated with JSH-23 (a p65 nuclear translocation inhibitor) for 24 hours. **B** Quantitative analysis of p65 and CUL3 derived from the data presented in Panel A. Data are presented as the mean ± SD (n = 3). ***p* < 0.01. WCL, whole cell lysate; NP, nuclear proteins. **C** Western blot analysis of CUL3 expression in HaCaT cells with *CUL3*-OE. Data are presented as mean ± SD (n=3), **p* < 0.05. **D** mRNA expression levels of chemokines (*CCL20, CXCL1, CXCL2*) and the antimicrobial peptide *S100A7* in HaCaT cells under various treatment conditions. Cells were transfected with a CUL3 overexpression plasmid (CUL3-OE) or a negative control (NC) for 24 hours, followed by stimulation with or without M5 for an additional 24 hours. Data are presented as the mean ± SD (n = 3). ***p* < 0.01, *** *p* < 0.001.

**Figure S7. Immunohistochemical staining of Ki-67 and keratin 1 in the IMQ-induced psoriasiform skin from mice treated with Scramble shRNA (sh-Sc) or *Gpr107* knockdown (sh-#1 and sh-#2) viral constructs.**

**A** Immunohistochemistry staining of Ki-67-positive cells (from Fig 7E). **B** Immunohistochemistry staining of keratin 1 (K1) (from Fig 7G). Scale bars: upper panels = 100 μm; lower panels = 200 μm.
